# Genome-wide association mapping and genomic prediction analyses reveal the genetic architecture of grain yield and agronomic traits under drought and optimum conditions in maize

**DOI:** 10.1186/s12870-025-06135-3

**Published:** 2025-02-01

**Authors:** Manigben Kulai Amadu, Yoseph Beyene, Vijay Chaikam, Pangirayi B. Tongoona, Eric Y. Danquah, Beatrice E. Ifie, Juan Burgueno, Boddupalli M. Prasanna, Manje Gowda

**Affiliations:** 1 International Maize and Wheat Improvement Center (CIMMYT), C/O: World Agroforestry Centre (ICRAF), United Nations Avenue, Gigiri, P.O. Box, Nairobi, 1041-00621 Kenya; 2 West Africa Centre for Crop Improvement (WACCI), University of Ghana, PMB 30 Legon, Accra, Ghana; 3 CSIR-Savanna Agricultural Research Institute, PO. Box 52, Tamale, Nyankpala Ghana; 4 International Maize and Wheat Improvement Center (CIMMYT), Km 45, Carretera México-Veracruz, El Batán, Edo. de Mexico CP 52640 Mexico; 5 Institute of Biological, Environmental & Rural Sciences (IBERS), Aberystwyth University, Aberystwyth, Wales SY23 3EE UK

**Keywords:** Maize, Yield, Drought, Genome-wide association study, Haplotype, Genomic prediction

## Abstract

**Background:**

Drought is a major abiotic stress in sub-Saharan Africa, impacting maize growth and development leading to severe yield loss. Drought tolerance is a complex trait regulated by multiple genes, making direct grain yield selection ineffective. To dissect the genetic architecture of grain yield and flowering traits under drought stress, a genome-wide association study (GWAS) was conducted on a panel of 236 maize lines testcrossed and evaluated under managed drought and optimal growing conditions in multiple environments using seven multi-locus GWAS models (mrMLM, FASTmrMLM, FASTmrEMMA, pLARmEB, pKWmEB, ISIS EM-BLASSO, and FARMCPU) from *mrMLM* and *GAPIT R packages.* Genomic prediction with RR-BLUP model was applied on BLUEs across locations under optimum and drought conditions.

**Results:**

A total of 172 stable and reliable quantitative trait nucleotides (QTNs) were identified, of which 77 are associated with GY, AD, SD, ASI, PH, EH, EPO and EPP under drought and 95 are linked to GY, AD, SD, ASI, PH, EH, EPO and EPP under optimal conditions. Among these QTNs, 17 QTNs explained over 10% of the phenotypic variation (*R*^*2*^ ≥ 10%). Furthermore, 43 candidate genes were discovered and annotated. Two major candidate genes, *Zm00001eb041070* closely associated with grain yield near peak QTN, *qGY_DS1.1* (*S1_216149215*) and *Zm00001eb364110* closely related to anthesis-silking interval near peak QTN, *qASI_DS8.2* (*S8_167256316*) were identified, encoding *AP2-EREBP* transcription factor 60 and TCP-transcription factor 20, respectively under drought stress. Haplo-pheno analysis identified superior haplotypes for *qGY_DS1.1* (*S1_216149215*) associated with the higher grain yield under drought stress. Genomic prediction revealed moderate to high prediction accuracies under optimum and drought conditions.

**Conclusion:**

The lines carrying superior haplotypes can be used as potential donors in improving grain yield under drought stress. Integration of genomic selection with GWAS results leads not only to an increase in the prediction accuracy but also to validate the function of the identified candidate genes as well increase in the accumulation of favorable alleles with minor and major effects in elite breeding lines. This study provides valuable insight into the genetic architecture of grain yield and secondary traits under drought stress.

**Supplementary Information:**

The online version contains supplementary material available at 10.1186/s12870-025-06135-3.

## Introduction

Maize (*Zea mays* L.) is an indispensable cereal crop in global agri-food systems[1, 2]. However, grain yield is stagnating due to unpredictable climate change and increase in negative impacts of drought on maize production and productivity in sub-Saharan Africa (SSA) [3–6]. Boosting maize grain yield potential and improving maize resilience to drought are key solutions proposed for mitigating the effects of drought and climate changes while minimizing farmer’s risk [7, 8]. In SSA, it is estimated that about 40% of the region’s maize-growing area experiences intermittent drought stress, leading to sizable yield reductions ranging from 10 to 25% [9, 10]. Maize plants require at least 500 to 800 mm of water, which translates to 5.6–6.7 mm per day. This largely depends on the maturity group, soil moisture, growth stage, and environmental conditions [11, 12]. Water deficit below this range can lead to severe water stress in maize plants, particularly during the flowering stage. The maize plants respond to drought stress by rolling their leaves, reducing leaf area, and closing stomata, which affects photosynthetic activity and enzyme production. Drought stress coinciding with the flowering and grain-filling stages in maize causes a sizable yield reduction of up to 90% [13–15].

Considerable efforts have been made to boost the grain yield potential and stress-resilience in maize through conventional breeding[3, 16, 17]. However, genetic improvement of grain yield under drought stress through conventional breeding methods has proven to be challenging [18], primarily caused by the multigenic nature of traits, controlled by many loci, each contributing a small effect[13, 19, 20]. Owing to its multigenic nature of inheritance and genotype x environment interactions, grain yield often presents low heritability under drought conditions [21]. For this reason, it is difficult to accurately estimate breeding values, which results in lower genetic gain per unit time and thereby constrains the development of drought-tolerant maize hybrids[22]. This, therefore, emphasizes the need to complement conventional breeding methods with genomic-assisted breeding tools to accelerate the development of high yielding drought-tolerant maize cultivars thereby boosting productivity in stress-prone areas.

Recent advances in crop genomics and phenomics have increased our understanding of the physiological and genetic basis of complex traits such as drought tolerance and grain yield [20, 23]. Linkage mapping based on biparental population is one of the most powerful tools extensively utilized to identify several quantitative trait loci (QTL) related to grain yield and secondary traits under drought stress [22, 24, 25]. However, many constructed maps suffer from low resolution and low allele richness [25]. Contrary to linkage mapping, GWAS utilizes diverse natural populations, which eliminates the need for developing segregating populations, saving time and cost[26] and hence is the most preferred method [27, 28]. Moreover, it can detect multi-allelic variation, rare, and small effect QTLs simultaneously [29], providing high resolution by leveraging historical meiotic recombination events available in diverse natural populations[30–32]. Conversely, the results of GWAS can be influenced by the population structure, which can significantly interfere with the power of QTL detection[31, 33]. For this reason, several statistical models have been developed including single-locus GWAS models and multi-locus GWAS models [33]. In single-locus GWAS models such as the general linear model (GLM) [34]; Mixed linear model (MLM)[35]; Enriched compressed mixed linear model (ECMLM)[36]; efficient mixed model association eXpedited (EMMAX) [37] and genome-wide efficient mixed-model association (GEMMA)[38], test marker-trait associations for significance by multiple testing one marker at a time. These models incorporate population structure (e.g. Principal component, Kinship matrices, etc.) as fixed covariates or a random polygenic effect to address the genetic relatedness among individuals in a diverse population [39] and tend to detect major QTLs. However, single locus models are often prone to high false positive rates or Type 1 errors. Bonferroni correction is commonly employed to control false positive rate (FPR)[40]. However, the use of Bonferroni correction has been proven to be too conservative such that true quantitative trait nucleotides (QTNs) may be missed out when considering SNPs in linkage disequilibrium (LD). Therefore, multi-locus GWAS models have been recommended for addressing multiple test corrections[41].

Multi-locus GWAS models do not require Bonferroni correction, have higher power for detection of both major and minor QTL effects, and have proven to be superior to single locus models in detecting small effect loci[40]. For this reason, several multi-locus GWAS including the multiple loci mixed model (MLMM), fixed and random model circulating probability unification (FarmCPU)[42], and Bayesian-information and linkage-disequilibrium iteratively nested keyway (BLINK)[43]. In addition, other key models are multi-locus random-SNP-effect mixed linear model (mrMLM)[44], the fast multi-locus random-SNP-effect mixed linear model (FASTmrMLM) [45], the fast multi-locus random-SNP-effect efficient mixed-model association (FASTmrEMMA) [46], polygenic-background-control-based least angle regression plus empirical Bayes (pLARmEB)[47], polygenic-background-control-based Kruskal–Wallis test plus empirical Bayes (pKWmEB) [48], and Iterative sure independence screening expectation maximization Bayesian least absolute shrinkage and selection operator (ISIS EM-BLASSO) [49] are commonly used now. The multi-locus GWAS methods involve a two-step process. In the first step, a single-dimensional genome scan is implemented using a less stringent critical value to identify putative QTLs. In the second step, all putative QTLs identified in the first step are subjected to further genome-wide scan to identify true QTNs using a logarithm of the odds (LOD) statistics to determine their significance[50].

Genomic selection (GS) or genomic prediction (GP) is another efficient genomic-assisted breeding tool in which genome-wide markers are fed into a prediction model to predict the genomic estimated breeding values (GEBVs) of lines in a breeding population [51]. It has enormous potential for improving drought tolerance in maize, as it allows for more accurate selection of complex traits such as grain yield under drought and reduces the breeding cycles by enhancing the genetic gain per unit time [52]. Several studies have demonstrated the effectiveness of utilizing GS for improving grain yield and secondary traits in drought tolerance breeding in maize[7, 53–55]. Zhang et al.[56] reported low to medium prediction accuracy for grain yield (GY) and secondary traits in maize under drought stress. The results indicated that several factors influenced the prediction accuracy values, including the types of breeding populations, the size of the training population, the complex nature of traits, the marker densities, and the genotyping platforms. Therefore, the combined application of different GWAS models and GS can enhance the power of QTL detection and accelerate breeding to improve drought tolerance and assist in selecting superior genotypes under drought stress.

The objectives of the study were to (1) identify significant QTNs and putative candidate genes for GY and secondary traits under optimum and drought stress (DS) using multi-locus GWAS models and (2) assess the potential of GS in improving GY and related traits under DS and optimum conditions. These results will further deepen our understanding of the genetic architecture of complex traits, especially for GY and drought tolerance, which is critical for making accurate selections and developing stress-resilient maize hybrids.

## Material and methods

### Germplasm, Experimental design, and Phenotyping

A panel of 236 maize inbred lines was assembled for this study. The lines were developed by International Maize and Wheat Improvement Centre’s (CIMMYT) Global Maize Program through conventional breeding and doubled haploid technology [57]. These elite maize lines were crossed with a popular single cross hybrid tester (CML566 x CML395) and produced 236 test cross hybrids. All test cross hybrids plus six commercial hybrids as checks (DHO4, DK8031, H513, PH3253, Pioneer 3253, and WH505) were evaluated under DS and optimum conditions. The optimum experiments were conducted under rainfed conditions which was augmented with irrigation to avoid DS at seven (7) locations, that is, Embu [0.48° S 37.47° E, 1159 masl], Kaguru [37.67°E,-0.08°S,1463masl], Kakamega [0.29°N, 34.77°E,1535 masl], Kiboko[37.72°E, 2.22°S, 975 masl], Kirinyaga [37.19`E, 0.34`S, 1282masl], Mwtapa [3.93° S 39.74° E, 30 masl] and Shikutsa [ 0.28° N, 34.75° E,1561 masl]. The drought experiment was conducted at three (3) locations, namely Kiboko [37.72°E, 2.22°S, 975 masl], Homabay [0.52°S, 34.45°E, 1751 masl] and Mtwapa [3.93°S, 39.74°E, 30 masl] during the dry season. The drought experiment was conducted following the protocol established by CIMMYT [19, 58]. The drought experiments were irrigated once a week using a drip irrigation system until two weeks before the expected flowering date. Irrigation was withdrawn to maintain DS until harvest.

The experiments were set up in a 5 × 49 alpha lattice design with two replications. The experimental unit was a two-row plot of 5 m long with intra-row spacing of 0.25 m and inter-row spacing of 0.75 m. Two seeds per hill were seeded and later thinned to one plant per hill three weeks after seedling emergence to a final plant population of 53,333 plants/ha. Basal fertilizer application was carried out at planting using di-ammonium phosphate (D.A.P) fertilizer at the rate of 60 kg N and 60 kg P_2_O_5_ per hectare. Six weeks after emergence, all experiments were top-dressed with Urea at the rate of 60 kg N. All the experiments were kept weeds-free by manual weeding and herbicide control. Detailed information on the pedigree of the inbred lines used in this study is presented in Supplementary Table S1.

### Phenotypic data collection and analysis

In all the experiments, data were collected on days to 50% anthesis (AD) and days to 50% silking (SD) as the number of days from planting to the day, when half of the plants per plot had shed pollen and silks emerged, respectively. Anthesis-silking interval (ASI) was computed as the difference between SD and AD. Plant height (PH) and ear height (EH) were measured in centimeters from the base of the plant to the height of the first tassel branch and the node bearing the upper ear, respectively. Ear position (EPO) was measured as the ratio of ear height to plant height per plot. The number of ears per plant (EPP) was determined by dividing the total number of ears per plot by the number of plants harvested per plot. Ears from each plot were shelled and weighed to determine grain yield (GY) in kilograms, which was then converted to tons per hectare (t/ha). Moisture content (MOI) of the shelled grains at harvest was measured with a portable handheld moisture meter and recorded in percentage. GY per plot in tons per hectare will be calculated using the field weight of harvested ears per plot and adjusted 12.5% moisture content. All trait measurements were done according to the procedure outlined in the drought phenotyping protocol of CIMMYT [58, 59].

Data was analyzed for each and across locations under optimum and DS conditions. The restricted maximum likelihood (REML) estimates of variance components, coefficient of variation, broad-sense heritability, phenotypic and genetic correlation among traits for individual and combined analysis (Eq. 1) were estimated using multi-environment trial analysis R package (META-R) [60]. The linear mixed models available in META-R were implemented using the *Lme4* R-package [61]. In the model, all factors were treated as random effects in this analysis except the genotype effect to estimate the best linear unbiased estimates (BLUEs). The best linear unbiased predictions (BLUPs) and variance components were estimated by treating all factors as random except replication and environments.

1 $${{\varvec{Y}}}_{{\varvec{i}}{\varvec{j}}{\varvec{k}}{\varvec{l}}}={\varvec{\mu}}+\boldsymbol{ }{{\varvec{E}}{\varvec{n}}{\varvec{v}}}_{{\varvec{i}}}+{{\varvec{R}}{\varvec{e}}{\varvec{p}}}_{{\varvec{j}}}\left({{\varvec{E}}{\varvec{n}}{\varvec{v}}}_{{\varvec{i}}}\right)+{{\varvec{B}}{\varvec{l}}{\varvec{o}}{\varvec{c}}{\varvec{k}}}_{{\varvec{k}}}\left({{\varvec{R}}{\varvec{e}}{\varvec{p}}}_{{\varvec{j}}}{{\varvec{E}}{\varvec{n}}{\varvec{v}}}_{{\varvec{i}}}\right)+{{\varvec{G}}{\varvec{e}}{\varvec{n}}}_{{\varvec{l}}}+\boldsymbol{ }{{\varvec{E}}{\varvec{n}}{\varvec{v}}}_{{\varvec{i}}}\boldsymbol{ }x\boldsymbol{ }{{\varvec{G}}{\varvec{e}}{\varvec{n}}}_{{\varvec{l}}}+\boldsymbol{ }+{{\varvec{\varepsilon}}}_{{\varvec{i}}{\varvec{j}}{\varvec{k}}{\varvec{l}}}$$

where ${{\varvec{Y}}}_{{\varvec{i}}{\varvec{j}}{\varvec{k}}{\varvec{l}}}$ is the trait of interest, ${\varvec{\mu}}$ is the overall mean effect; ${{\varvec{E}}{\varvec{n}}{\varvec{v}}}_{{\varvec{i}}}$ is the effect of ${i}^{th}$ environment;${{\varvec{R}}{\varvec{e}}{\varvec{p}}}_{{\varvec{j}}}\left({{\varvec{E}}{\varvec{n}}{\varvec{v}}}_{{\varvec{i}}}\right)$ is the effect of the ${j}^{th}$ replicate nested within the ${i}^{th}$ environment; ${{\varvec{B}}{\varvec{l}}{\varvec{o}}{\varvec{c}}{\varvec{k}}}_{{\varvec{k}}}\left({{\varvec{R}}{\varvec{e}}{\varvec{p}}}_{{\varvec{j}}}{{\varvec{E}}{\varvec{n}}{\varvec{v}}}_{{\varvec{i}}}\right)$ is the effect of ${k}^{th}$ incomplete block nested within ${j}^{th}$ replicate in ${i}^{th}$ environment; ${{\varvec{G}}{\varvec{e}}{\varvec{n}}}_{{\varvec{l}}}$ is effect of ${l}^{th}$ genotype; ${{\varvec{E}}{\varvec{n}}{\varvec{v}}}_{{\varvec{i}}}\boldsymbol{ }x\boldsymbol{ }{{\varvec{G}}{\varvec{e}}{\varvec{n}}}_{{\varvec{l}}}$ is the effect of genotype x environment interactions and ${{\varvec{\varepsilon}}}_{{\varvec{i}}{\varvec{j}}{\varvec{k}}{\varvec{l}}}$ is the residual effect. The variance components from the combined analysis were used to compute broad sense heritability [62].

### DNA extraction, Sequencing, SNP discovery, and calling

Genomic Deoxyribonucleic acid (DNA) of 236 lines was extracted from seedlings at the 4-leaf stage using a modified version of CIMMYT’s high throughput mini-prep Cetyl Trimethyl Ammonium Bromide (CTAB) protocol [63]. The DNA samples were shipped to Cornell University for genotyping. In brief, the high-quality DNA extracted from each leaf sample of the 236 lines was digested with restriction endonuclease *Ape KI.* DNA libraries were constructed for each sample and sequenced using genotyping-by-sequencing (GBS) protocol as described by Elshire et al. [64]. Raw GBS data of 955,690 SNPs distributed across the ten maize chromosomes were received from the Institute of Biotechnology at Cornell University, USA, after mapping to B73 AGPv2 coordinates. SNP calling was carried out using the TASSEL-GBS pipeline [65]. The raw GBS data were cleaned by removal of SNP markers with a minimum count of 90%, greater than 5% heterozygosity, and less than 5% minor allele frequency using TASSEL software version 5.2 [66], resulting in a total of 230,743 SNPs. In addition, the lines with greater than 20% missing data and SNPs not located on any of the ten chromosomes were further filtered out to a final dataset of 215,542 SNPs in 236 diverse lines for further analysis. The density and distribution map of SNPs on each of the ten maize chromosomes was drawn using a *CMplot* R package (https://github.com/YinLiLin/CMplot).

### Population structure and linkage disequilibrium analysis

To capture population structure and cryptic genetic relatedness among the 236 lines, population structure, and kinship analysis were carried out using 215,542 genome-wide SNPs distributed across the ten chromosomes. The population structure was estimated using the admixture model method implemented in the software package STRUCTURE version 2.3.4 [67, 68]. The number of subpopulations (K) was set from 1 to 15 with 10 independent runs for each K. The burn-in length and Markov Chain Monte Carlo (MCMC) replication were set at 100,000 each run under the admixture and correlated allele frequency model. The STRUCTURE HARVESTER [69], a web-based program was used to summarize STRUCTURE output, visualize the likelihood values across multiple values of *K* and, compute the natural logarithms of probability data *[LnP(K)*] and the ad hoc statistic *ΔK* based on *Evanno* method [70].

The principal component analysis (PCA) was conducted using the Genomic Association and Prediction Integrated Tool (GAPIT) version 3[71, 72] to detect the subpopulation structure present in the panel. The kinship matrix was estimated using the VanRaden algorithm [73] to measure the genetic relatedness among individuals in the association panel. The genetic relationship among the lines was determined based on the neighbor joining tree algorithm using the phylogenetic tree analysis in TASSEL software v5.2.93 [66]. To determine the extent of linkage disequilibrium (LD), squared allele frequency correlations (*r*^*2*^) between all pairs of SNP markers were estimated using TASSEL software version 5.2.93 [66]. To calculate the LD decay rate, the nonlinear regression model developed by [74], with modifications by Remington et al. [75], was used to fit the LD decay curve into the scatterplot using the LOESS function in R.

### Genome-wide association study

GWAS analysis was carried out with 215,542 high quality SNPs (G) from 236 lines with BLUP values of eight phenotypic traits (GY, AD, SD, ASI, EPO, EPP, PH, and EH) using different multi-locus (ML) GWAS models under DS and optimum conditions. The first four PCAs and kinship (K) matrix were incorporated in the GWAS models as covariates to reduce false positives. The ML-GWAS was conducted with seven models including: (1) Fixed and random model circulating probability unification (FarmCPU)[42], (2) mrMLM[44], (3) FASTmrMLM [45], (4) FASTmrEMMA [46], (5) pLARmEB[47], (6) pKWmEB[48], and (7) ISIS EM-BLASSO[45]. One multi-locus model was implemented in (GAPIT) R package software[71, 72] and the other six multi-locus models were implemented in the *mrMLM* R package[40]. The nomenclature for naming QTN was done using the letter “q” to indicate QTN, followed by an abbreviation representing the trait name, underscoring the management conditions, the corresponding chromosome number, and the number of QTNs identified on that specific chromosome.

To determine the genome-wide significant *P* values threshold for the FarmCPU GWAS results, the effective number of independent SNPs (N) were calculated using the SimpM R program[30, 76, 77] available on GitHub (https://github.com/LTibbs/SimpleM). The genome-wide significant *P* value threshold was adjusted based on Bonferroni correction as the ratio of alpha value (*α* = 0.05) divided by the effective number of independent SNPs (*N* = 79,455). Hence, the genome-wide significant and suggestive levels were set as *P* = 0.05/*N* = 6.29 × 10^−7^ and *P* = 1/*N* = 1.26 × 10 − 5, respectively, where *N* is the effective number of independent SNPs. For multi-locus GWAS analysis, the genome-wide significant threshold was defined based on the threshold of LOD ≥ 3 (*p* = 0.0002). Manhattan plots and quantile–quantile plots were developed to visualize GWAS results using *CMplot R* package (https://github.com/YinLiLin/CMplot).

### Candidate gene annotation and haplotype block analysis

The LD decay with a physical distance of 4.75 kb found in this study was used to find candidate genes. All the candidate genes for GY, AD, SD, PH, EH, EPO, and EPP located within regions from 4.75 kb upstream to 4.75 kb downstream associated with significant QTNs were identified and annotated using the B73 maize reference genome (B73 RefGen_V2)[78–80]. The candidate gene annotations information was retrieved from the maizeGDB database (http://www.maizegdb.org).

The significant QTN, *qGY_DS1.1* (S1_216149215) on chromosome 1 for GY located within the genomic regions of a candidate gene, *Zm00001eb041070* were extracted from the variant call format (VCF) using the site filtering option of VCF tools [81]. The haplotype block analysis was then implemented in Haploview software version 4.2 [82] and *geneHapR* [83]*.* The blocks were defined according to the criteria described by Gabriel et al. [84]. One-way Analysis of variance (ANOVA), boxplot, and multiple comparisons of phenotypic differences among haplotypes were implemented in *agricolae R package* using Tukey’s Honestly Significant differences (HSD) test.

### Genomic-wide prediction

GP was carried out on the 236 lines based BLUE values for traits across environments within management using ridge regression best linear unbiased prediction (*rrBLUP*) model available in the *rrBLUP R package* [85]. Genomic estimated breeding values (GEBVs) were estimated using a five-fold cross-validation scheme by randomly sampling 80% and 20% of maize lines as training and testing sets, respectively. The prediction accuracy of the model was computed as the average Pearson’s correlation coefficient (r) between GEBV estimates from the training and testing set with 100 iterations. Bar plot was generated for each trait to visualize the means and standard deviation of prediction accuracy using the *ggplot2 R package* [86].

## Results

### Phenotypic variation, Descriptive statistics, and Correlation

**Fig. 1:**
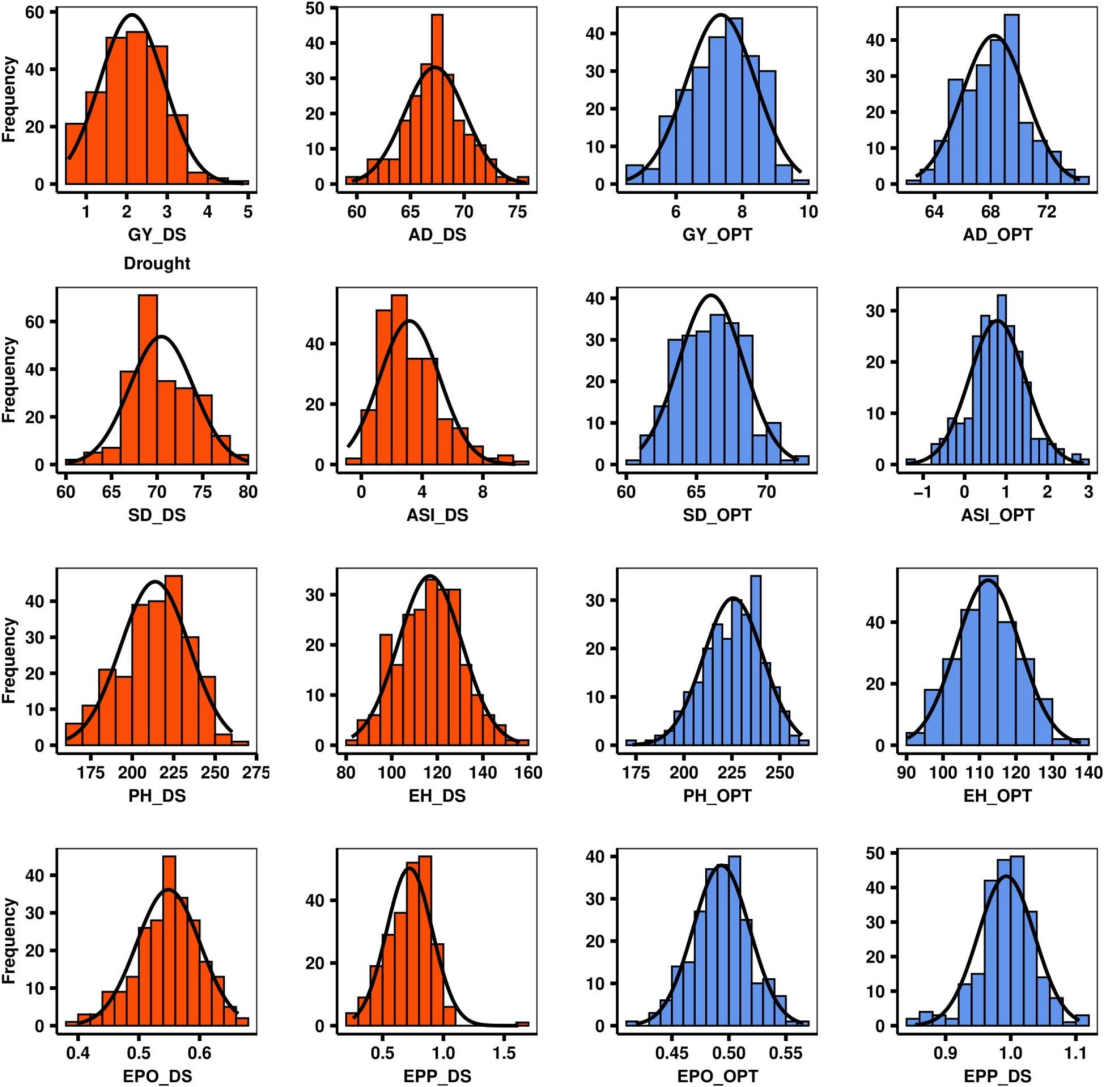
Frequency distributions for GY (grain yield); AD (days to 50% anthesis); SD (days to 50% silking); ASI (Anthesis-Silking interval); EPO (Ear position, Ear–plant height ratio), EPP (Ear per plant, PH = Plant height. and EH (Ear height). in 236 diverse maize lines evaluated under drought and optimum conditions. Underscore DS = Drought and OPT = Optimum conditions

The combined analysis of variance revealed significant (*p* < 0.05) genotype and GEI variations for GY, AD, SD, ASI, EPO, EPP, EH, and PH under DS and optimum conditions (Table 1). The mean, minimum, and maximum values of GY, AD, SD, ASI, EPO, EPP, EH, and PH revealed large variability for each trait under both DS and optimum conditions (Table 1). GY under DS was reduced by 71%. PH (214.69 cm) were reduced significantly under DS compared to the mean of PH (225.94 cm). Whereas EH (116.88 cm) were relatively increased under DS compared to mean EH (112.27 cm) under optimum conditions.DS had a relatively small effect on EPO and EPP. DS conditions increased the average number of days for AD and SD compared to optimum conditions. Consequently, prolonging the interval between AD and SD (known as ASI) under drought by an average of 3 days compared to optimum conditions. The broad-sense heritability ranged from 0.25 for GY to 0.87 for EPO under DS and varied from 0.21 for EPP to 0.74 for AD under optimum conditions. The frequency distribution of each of the traits under the two experimental conditions (DS and optimum) is shown in Fig. 1 where most of the traits showed continuous distributions.

**Table 1:** Descriptive statistics, analysis of variance, broad sense heritability, least significant difference (LSD) and coefficient of variation of agronomic traits under drought and optimal conditions

Statistic	Min	Max	Mean ± SD	${\sigma }_{G}^{2}$	${\sigma }_{GE}^{2}$	${\sigma }_{e}^{2}$	${H}^{2}$	LSD_5%_	CV (%)
**Drought Conditions**									
GY	1.08	3.84	2.13 ± 0.47	0.06^b^	0.28^b^	0.54	0.25	1.85	22.23
AD	60.56	77.11	67.56 ± 2.33	4.71^b^	0.97^b^	2.88	0.85	3.10	2.69
SD	63.31	76.98	70.36 ± 2.36	4.98^b^	1.68^b^	5.38	0.77	3.85	3.60
ASI	0.56	6.64	2.77 ± 1.02	0.49^a^	0.69^b^	2.17	0.46	1.64	95.88
EPO	0.42	0.64	0.55 ± 0.04	0.002^b^	0.0003^b^	0.00	0.87	0.05	5.94
EPP	0.50	1.13	0.75 ± 0.08	0.0031	0.01^b^	0.03	0.28	0.14	19.72
PH	174.71	245.72	214.69 ± 12.82	140.23^b^	56.48^b^	260.31	0.24	22.72	8.58
EH	86.41	145.30	116.88 ± 10.33	86.29^b^	28.48^b^	103.41	0.69	16.12	11.02
**Optimal Conditions**									
GY	6.05	8.51	7.38 ± 0.48	0.44^b^	0.54^b^	2.94	0.46	1.36	23.18
AD	61.57	69.82	65.27 ± 1.64	3.82^b^	1.74^b^	7.47	0.74	2.87	4.18
SD	62.85	70.13	66.04 ± 1.50	3.37^b^	1.65^b^	9.91	0.67	2.99	4.77
ASI	0.33	1.77	0.74 ± 0.33	0.21^b^	0.13^b^	1.44	0.49	0.91	13.10
EPO	0.44	0.54	0.49 ± 0.02	0.0004^b^	0.0005^b^	0.00	0.67	0.03	5.30
EPP	0.97	1.03	1.00 ± 0.01	0.001^b^	0.002^b^	0.01	0.21	0.06	11.53
PH	192.18	249.47	225.94 ± 9.77	139.39^b^	117.15^b^	369.56	0.65	19.86	8.49
EH	97.96	129.19	112.27 ± 5.60	53.22^b^	58.49^b^	104.82	0.66	12.18	9.13

*¥Min* Minimum value, *Max* Maximum value, *SD* Standard deviation,${\upsigma }_{\text{G}}^{2}$Genetic variance,${\upsigma }_{\text{GE}}^{2}$Genotype by environment interactions variance,${\sigma }_{e}^{2}$Residual variance,${H}^{2}$Broad sense heritability, *LSD* Least Significant Difference, *CV* Coefficient of variation, *GY* Grain yield, *AD* Days to 50% anthesis, *SD* Days to 50% silking, *ASI* Anthesis-silking interval, *EPO* Ear position, *EPP* Ear per plant, *PH* Plant height, *EH* Ear height

^a^Significant at 0.05 probability level

^b^Significant at 0.01 probability level

**Fig. 2:**
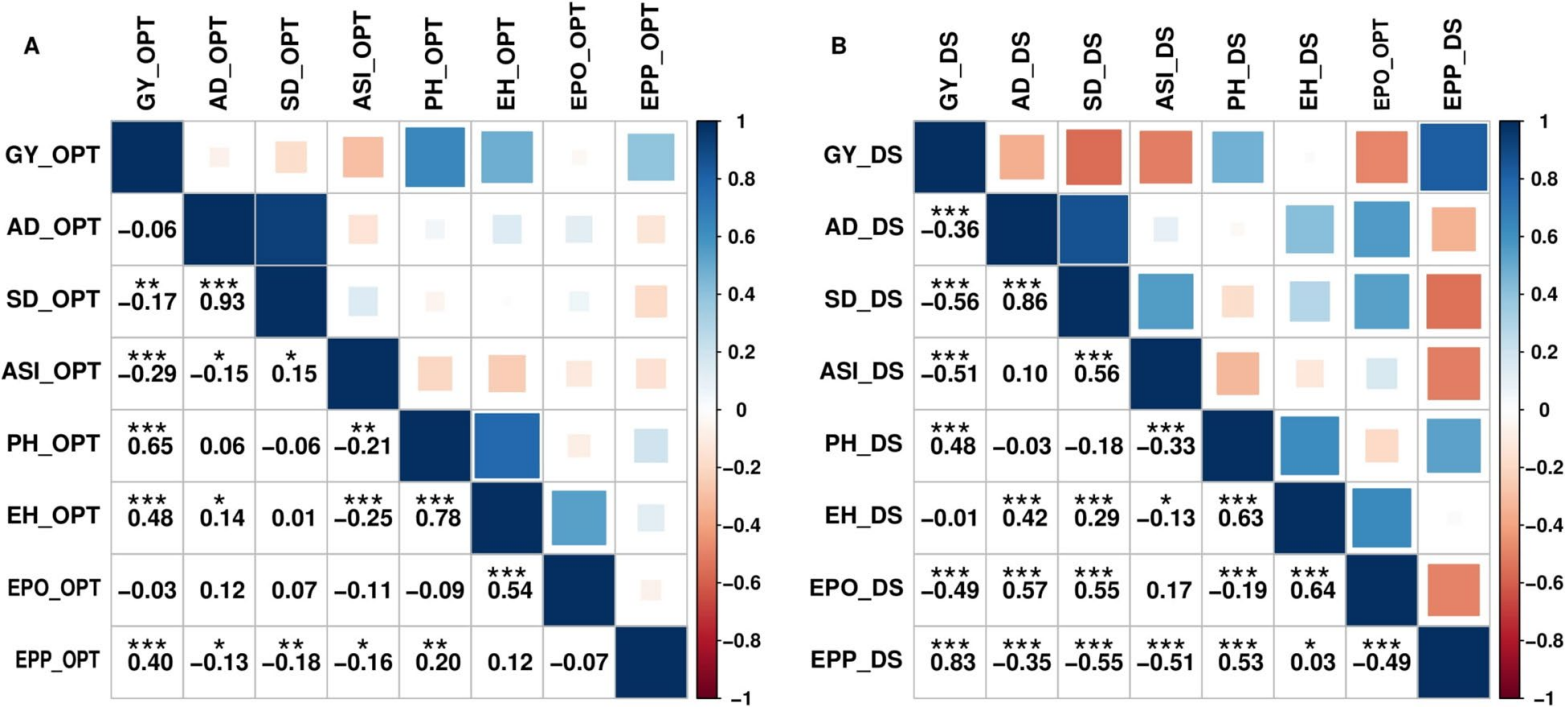
Pearson’s correlation analysis between the eight traits in 236 diverse maize lines under drought and optimum conditions. The blue color indicates the significant positive correlations, and red color indicates the significant negative correlation among different traits: GY (grain yield); AD (days to 50% anthesis); SD (days to 50% silking); ASI (Anthesis-Silking interval); EPO (Ear position, Ear–plant height ratio), EPP (Ear per plant, PH = Plant height. and EH (Ear height). (**A**) Under optimum (OPT) condition, (**B**) Under Drought (DS) condition

Pearson’s correlation analysis showing the relationships among traits under DS and optimum conditions is presented in Fig. 2. The correlation coefficients among the eight traits ranged from -0.29 to 0.93 under optimum conditions, whereas under DS conditions ranged from -0.56 to 0.86. Under optimum conditions, the highest significant positive correlation (r = 0.93**) was observed between flowering traits, SD_OPT and AD_OPT, followed by PH_OPT and EH_OPT (r = 0.78**), GY_OPT and PH_OPT (r = 0.65**). GY_OPT had a significant positive correlation with PH_OPT, EH_OPT, and EPP_OPT while a significant but negative correlation was observed between GY_OPT with SD_OPT and ASI_OPT. Similarly, ASI_OPT had a significant negative correlation with AD_OPT, PH_OPT, EH_OPT, and EPP_OPT. Under DS conditions, the highest positive correlations were 0.83 (between GY_DS and EPP_DS), 0.66 (SD_DS and AD_DS), 0.64 (EPO_DS and EH_DS), 0.63 (PH_DS and EH_DS), and 0.57 (EPO_DS and AD_DS). GY_DS was negatively and significantly correlated with AD_DS, SD_DS, ASI_DS, and EPO_DS, while positively and significantly correlated with PH_DS and EPP_DS. It was observed that ASI_DS was positively correlated with AD_DS, SD_DS, and EPO_DS and negatively correlated with PH_DS, EH_DS, and EPP_DS. Additionally, AD_DS was positively correlated with SD_DS, EH_DS and EPO_DS, but negatively correlated with PH_DS and EPP_DS. Overall, a strong correlation was observed between the AD and SD under both optimum and DS conditions.

### Marker distribution, Population structure, Phylogenetic tree, and Kinship

**Fig. 3:**
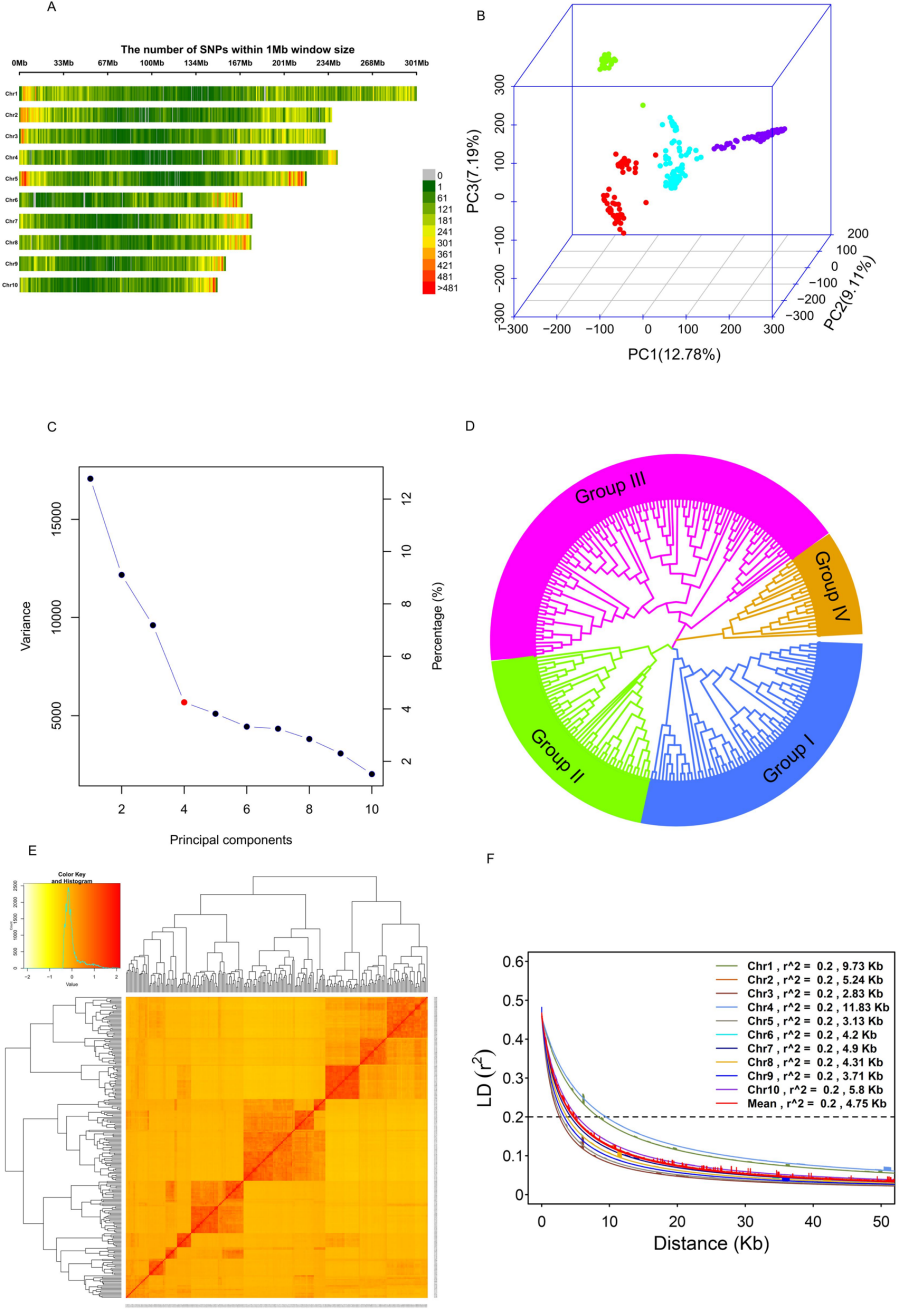
SNP density, Population Structure, and linkage disequilibrium (LD) in 236 diverse maize lines. **A** The distribution of 215,542 SNPs across the ten maize chromosomes. **B** Three-dimensional (3D) principal component analysis, displaying four distinct subpopulations (green, red, cadet blue, and blue). **C** A scree plot shows variance explained by each of the first ten principal components. The Optimal number of PCs retained, k = 4, is indicated in red dot. **D** Phylogenetic trees, displaying four unique groups based on neighbor-joining method. **E** A heat map of kinship matrix, showing the degree of genetic relatedness among the diverse maize lines. **F** Genome-wide average LD decay estimated across and for each of the ten maize chromosomes

The distribution of 215,542 SNPs across the genome is presented in Fig. 3A and supplementary Table S2. The number of SNP markers on each chromosome ranged from 14,629 to 33, 874 SNPs. Chromosome 1 had the highest number of SNPs of 33, 874, whereas chromosome 10 had the lowest number of SNPs with 14,629. The density of SNPs per mega-base pair (Mbp) varied from 84.89 Mbp for chromosome 4 to 116.79 Mbp for chromosome 5 with a mean of 104.28 Mbp.

The population structure of 236 diverse maize lines was determined by Bayesian based model in STRUCTURE and PCA (Supplementary Figure S1a and Fig. 3B). The optimum number of K was obtained by plotting the number of clusters (K) against delta K (Supplementary Figure S1a). The Bayesian structure analysis revealed the presence of three distinct subgroups within the 236 maize lines when K = 3 and ten distinct subgroups K = 10 (Supplementary Figure S1b). The genetic structure was further examined by PCA (Fig. 3B). The results revealed the presence of four distinct subpopulations separated by PC1 (12.78%), PC2 (9.11%), and PC3 (7.19%). The first three PCs explained over 29% of the total genetic variation among the diverse maize lines. A scree plot of variance explained against the corresponding PCs as shown in Fig. 3C was used to determine the optimal number of PCs to retain. The scree plot revealed that an optimal number (K) of four PCs could be retained for GWAS. The phylogenetic tree based on the neighbor-joining method as shown in Fig. 3D revealed that the 236 diverse maize lines can be clustered into four main groups (I = 66, II = 48, III = 100, and IV = 22) differentiated by the different colors (Supplementary Table S1).

The kinship matrix is utilized to assess the relatedness among individuals by considering the extent of allele sharing. The pattern of red shading in the center of the kinship matrix (Fig. 3E)) reflected the level of genetic relatedness among individuals, indicating the presence of four stratified population structures. In general, the results from the PCA, phylogenetic tree, and kinship matrix, show that the panel of 236 diverse maize lines can be divided into four subpopulations.

### Linkage Disequilibrium and GWAS analysis

The nonrandom association of alleles between two genetic loci was examined to provide valuable information in locating genes tightly linked to SNP markers associated with traits of interest. A rapid LD decay pattern was observed across the ten chromosomes (Fig. 3F). At *r*^*2*^ = 0.2, the LD decay distance between SNPs on the 10 chromosomes varied from 2.83 kb (Chr3) to 11.83 kb (Chr4) with an average genome-wide LD decay with physical distance between SNPs of 4.74 kb.

**Fig. 4:**
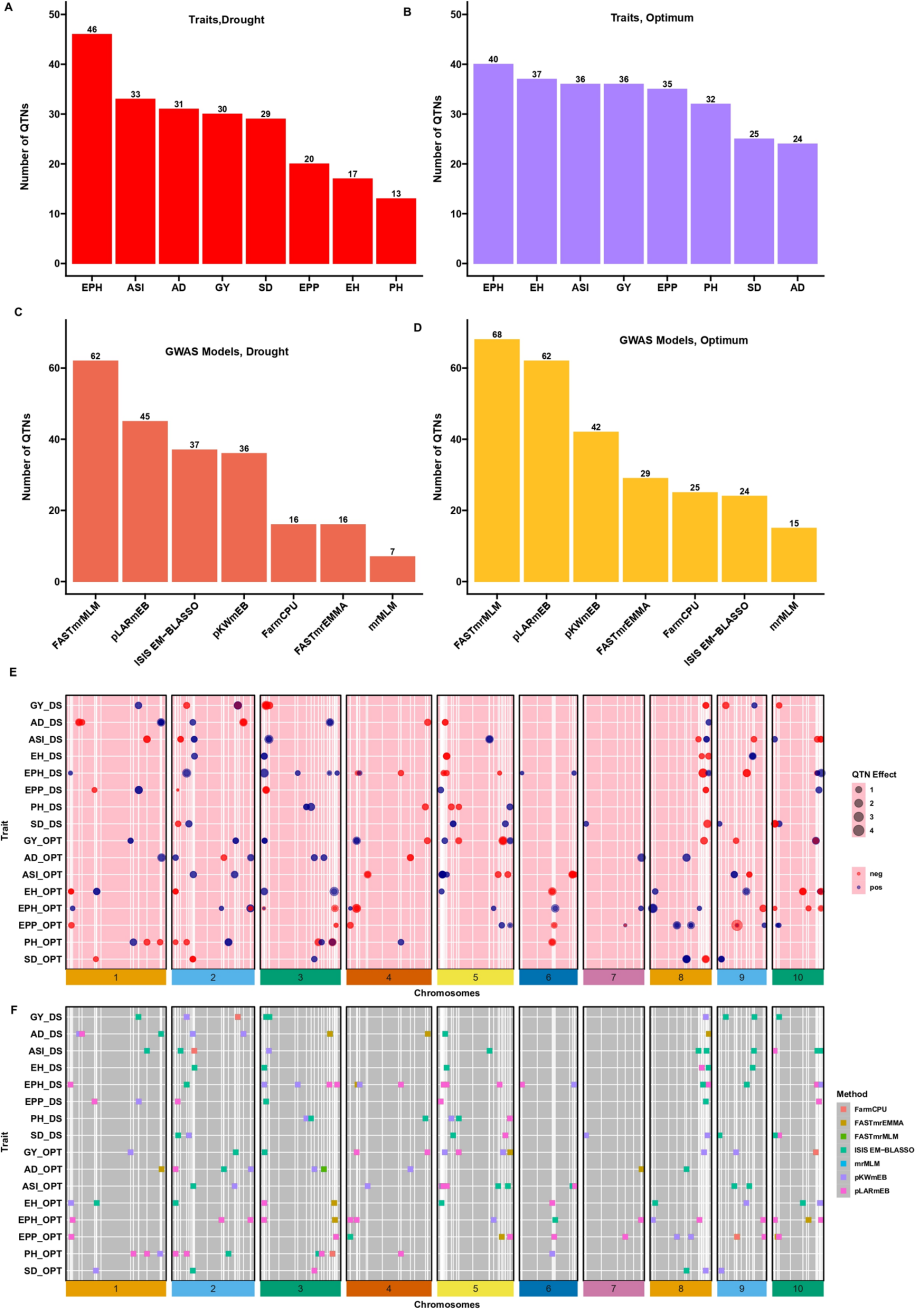
Number of significantly associated QTNs detected by each of the seven GWAS models implemented in this study. **A **Number of QTNs/SNP detected for grain yield (GY); days to 50% anthesis (AD); days to 50% silking (SD); Anthesis-Silking interval (ASI); plant height (PH), eight height (EH), ear-plant height ratio (EPH) and ear per plant (EPP) under drought condition (**B**). Number of QTNs/SNP detected for grain yield (GY); days to 50% anthesis (AD); days to 50% silking (SD); Anthesis-Silking interval (ASI); plant height (PH), eight height (EH), ear-plant height ratio (EPH) and ear per plant (EPP) under optimum condition **C**. Number of QTNs detected by the eight GWAS models under drought condition (**D**) Number of QTNs detected by the eight GWAS models under Optimum condition (**E**) Chromosomal Distribution of QTN effects. The circle diameter is proportional to the absolute value of the QTN effect. The colors indicate the direction of the effects: red indicates negative QTN effect, and blue indicates positive QTN effect. **F** Chromosomal distribution of QTNs based on seven GWAS methods. The x-axis indicates genomic locations by chromosomal order, and the significant QTNs are plotted against genome location. Each row represents one QTN identified by a different method

Based on the seven multi-locus GWAS models (FarmCPU, mrMLM, FastmrMLM, FASTmrEMMA, pLARmEB, pKWmEB and ISIS EM-BLASSO), 219 and 265 QTNs distributed across the 10 chromosomes were detected under DS and optimum conditions, respectively (Fig. 4, Supplementary Table S3 and S4). The QQ-plots depicting the observed vs expected LOD [− log10 (*p*), *p* = 6.29e-07] distributions showed a significant deviation from the expected distribution (Supplementary Figure S2 and S3), indicating the presence of QTNs linked to GY, AD, SD, ASI, PH, EH, EPP and EPO, respectively under DS and optimum conditions. The Manhattan plot showed that several QTNs with Bonferroni-adjusted threshold of greater than 6.20 [− log10 (*p*), *p* = 6.29e-07] and LOD threshold of ≥ 3 (*p* = 0.0002), were associated with GY, AD, SD, ASI, PH, EH, EPP and EPO, respectively under DS and optimum conditions.

The number of significant QTNs detected by multiple GWAS models varied across traits. Of these, the power of detection among the seven models followed FASTmrMLM (62 QTNs) > pLARmEB (45 QTNs) > ISIS EM-BLASSO (37 QTNs) > pKWmEB (36 QTNs) > FarmCPU (16 QTNs) > FASTmrEMMA (12 QTNs) > mrMLM (7 QTNs) under DS and FASTmrMLM (68 QTNs) > pLARmEB (62 QTNs) > pKWmEB (42 QTNs) > FASTmrEMMA (29 QTNs) > FarmCPU (25 QTNs) > ISIS EM-BLASSO (24 QTNs) > mrMLM (15 QTNs) under optimum conditions (Fig. 4C and 4D). Of these QTNs, 46, 33, 31, 30, 29, 20, 17, and 13 were found to be associated with EPO, ASI, AD, GY, SD, EPP, EH and PH under DS, respectively. Under optimum conditions, 40, 37, 36, 36, 32, 25, and 24 QTNs were associated with EPO, EH, ASI, GY, EPP, PH, SD and AD, respectively (Fig. 4A and 4B). With the proportion of *R*^2^ (%) by individual QTNs accounting for 0.00 ~ 8.18, 0.60 ~ 16.06, 3.93 ~ 15.29, 1.43 ~ 30.68, 0.00 ~ 11.42, 0.75 ~ 10.08, 1.32 ~ 16.06 and 2.00 ~ 12.66 (%) of total phenotypic variation for EPO, EH, ASI, GY, EPP, PH, SD and AD, respectively (Supplementary Table S4, S5 and S6).

Significant QTNs detected by at least two ML-GWAS models were considered reliable and stable. A sum of 172 QTNs comprising 77 QTNs under DS and 95 QTNs under optimum conditions were significantly associated with EPO, ASI, AD, GY, SD, EPP, EH, and PH, respectively (Fig. 4E and 4F; Supplementary Table S6). Of these significant QTNs under DS, 9, 9, 9, 11, 6, 6, 7, and 20 reliable QTNs were detected for GY, AD, SD, ASI, PH, EH, EPP, and EPO, respectively. The highest *R*^*2*^ (%) value was observed for GY QTN *qGY_DS2.1* [S2_194907656:*R*^*2*^ = 5.07%-11.07%], for AD *qAD_DS1.2* [*S1_32228028*: *R*^*2*^ = 5.27%-11.27%], for SD *qSD_DS10.1* [*S10_2032146*:*R*^*2*^ = 5.36%—9.92%], for ASI *qASI_DS5.1* [*S5_151886720*:*R*^*2*^ = 5.94%-11.19%], for PH *qPH_DS3.2* [*S3_149683959*:*R*^*2*^ = 9.62%-11.27%], for EH *qEH_DS2.1* [*S2_60655022*:*R*^*2*^ = 6.23%-6.78%], for EPP *qEPP_DS1.1* [*S1_217115118*:*R*^*2*^ = 9.19%-13.26%] and for EPO *qEPH_DS2.1* [*S2_36458273*:*R*^*2*^ = 0.96%—6.15%].

**Fig. 5:**
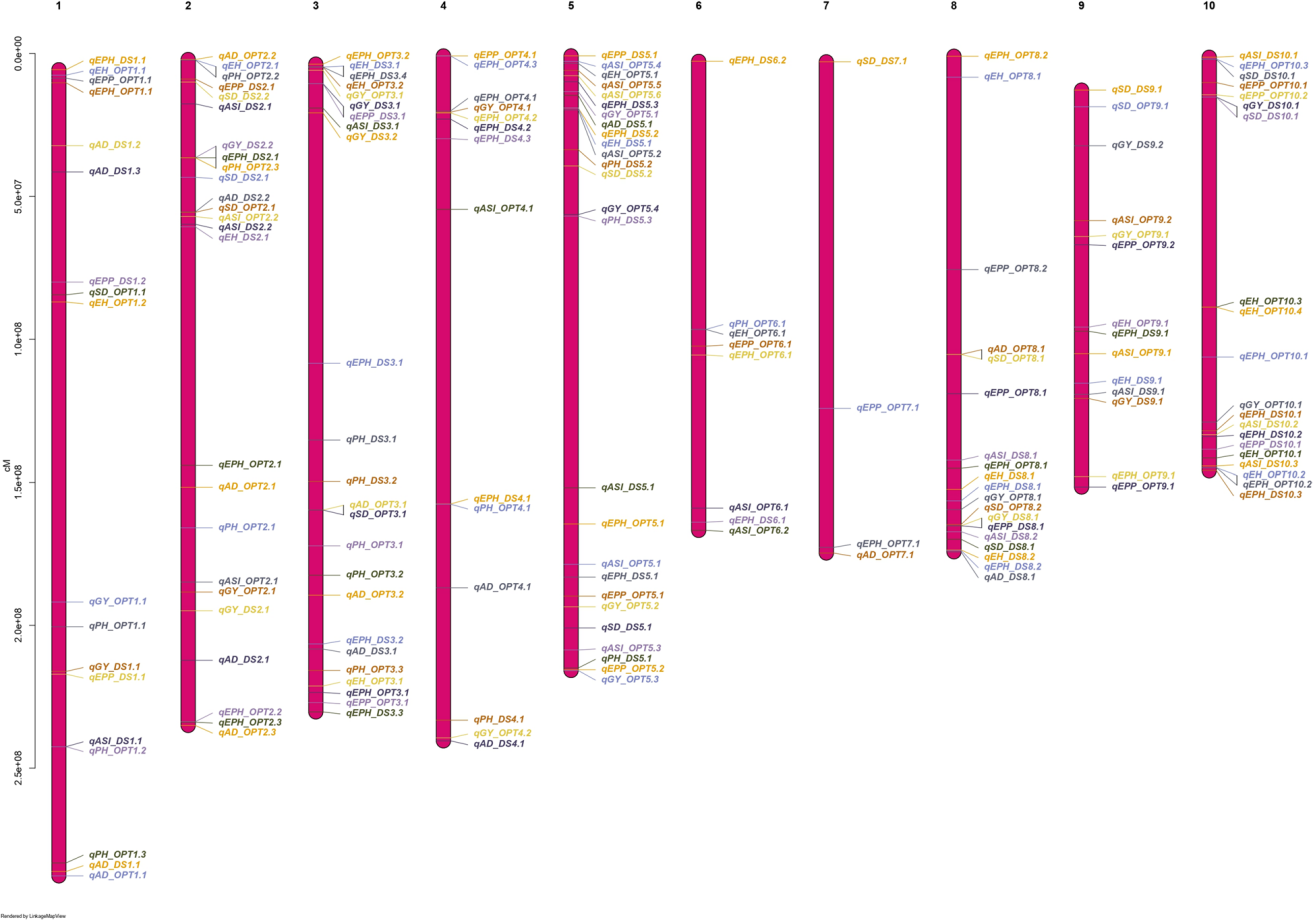
Chromosomal distribution of QTNs identified in this study

In addition, of the 95 QTNs detected in optimum condition, 12, 9, 6, 13, 13, 11, 18, and 13 QTNs were found to be associated with GY, AD, SD, ASI, EH, PH, EPO, and EPP, respectively. The QTN, *qGY_OPT5.2* [*S5_193538664*: *R*^*2*^ = 5.34%—10.19%] recorded the highest *R*^*2*^ (%) for GY. One QTN, *qAD_OPT8.1* (*S8_105322358*) was shared by AD (*R*^*2*^ = 6.03% -12.66%) and SD (*R*^*2*^ = 3.99%-16.06%). For ASI, *qASI_OPT6.1* [*S6_159006932*: *R*^*2*^ = 6.30%—14.10%] explained the highest *R*^*2*^ (%). While *qEH_OPT10.4* [*S10_88760138*: *R*^*2*^ = 9.39%—16.06%] showed the highest *R*^*2*^ (%) for EH, *qPH_OPT3.1* [*S3_172201680*: *R*^*2*^ = 1.49%—10.08%] for PH, *qEPO_OPT2.2* [*S2_233748945*: *R*^*2*^ = 1.56%—3.26%] for EPO and *qEPP_OPT8.2* [*S8_75600223*: *R*^*2*^ = 0.002% -5.48%] for EPP (Figs. 4 and 5).

The detected QTNs contributing revealed positive and negative allelic effects for different traits (Figs. 4E and 5). Among the 172 QTNs, few QTNs were detected under both DS and optimum conditions. For instance, two QTNs *qGY_DS2.1* and *qGY_DS1.1* explained the highest and positive effects with 0.34 and 0.24, respectively under DS for GY. On the other hand, two new QTNs *qGY_OPT4.1* and *qGY_OPT10.1* showed the highest and positive effects of 0.56 and 0.49, respectively for GY under optimum conditions. Negative QTN effects are desirable for flowering traits (AD, SD, and ASI) under DS for selecting earliness. For instance, QTNs *qAD_DS2.1*, *qSD_DS8.1*, *qASI_DS1.1* exhibited large and negative effects for AD (-1.72), SD (-1.79), and ASI (-0.77) on chromosome 2, 8 and 1, respectively (Fig. 4E). The distribution of QTNs (Fig. 5) on the 10 chromosomes showed that chromosome 5 captured the higher number of reliable QTNs (12 under DS and 14 under optimum conditions). Two QTNs, *qPH_OPT2.2* and *qEH_OPT2.1* exhibited a pleiotropic effect on both PH and EH under optimum conditions.

### Candidate gene identification and Annotation

Candidate genes analysis was conducted for significant QTNs identified in this study to elucidate the molecular, biological, and physiological mechanisms controlling traits under DS and optimum conditions. A total of 43 candidate genes were discovered and annotated, among them 18 and 25 candidate genes were identified under DS and optimum conditions, respectively (Table 2). Two candidate genes closely associated with the QTNs for improved GY were identified under DS, namely, *Zm00001eb041070* and *Zm00001d045665* (Table 2). Nine candidate genes potentially associated with flowering traits (AD, SD and ASI) were identified under DS (Table 2). Of these five candidate genes were associated with ASI under DS. Similarly, four and three candidate genes were found to be associated with EPO and EPP, respectively. Under optimum conditions, 3, 5, 8, 3, and 3 candidate genes were identified for GY, PH, EH, EPO, and EPP, respectively (Table 2). On the other hand, one candidate gene each was identified for AD, SD, and ASI.

**Table 2:** The candidate genes identified in association mapping panel for grain yield, flowering traits, plant and ear height, ear position and ear per plant under drought and optimum conditions

Trait	QTN	SNP	Chrom	Position	Gene_name	Annotation
**Drought Conditions**						
GY	*qGY_DS1.1*	S1_216149215	1	216,149,215	*Zm00001eb041070*	AP2-EREBP transcription factor 60
GY	*qGY_DS9.2*	S9_32284156	9	32,284,156	*Zm00001d045665*	Ubiquitin receptor RAD23c
AD	*qAD_DS1.1*	S1_286187136	1	286,187,136	*Zm00001eb058200*	Rubisco LSMT substrate-binding domain-containing protein
AD	*qAD_DS1.2*	S1_32228028	1	32,228,028	*Zm00001eb010210*	plant U-box type E3 ubiquitin ligase21
AD	*qAD_DS1.3*	S1_41437741	1	41,437,741	*Zm00001eb012590*	Trifunctional UDP-glucose 46-dehydratase
SD	*qSD_DS10.1*	S10_2032146	10	2,032,146	*Zm00001d023278*	Mad3/BUB1 homolog region 1
ASI	*qASI_DS1.1*	S1_242493792	1	242,493,792	*Zm00001eb047160*	kaurene synthase1
ASI	*qASI_DS8.2*	S8_167256316	8	167,256,316	*Zm00001eb364110*	TCP-transcription factor 20
ASI	*qASI_DS10.3*	S10_144315228	10	144,315,228	*Zm00001eb430770*	Ribosomal protein s11-beta
ASI	*qASI_DS10.2*	S10_133367573	10	133,367,573	*Zm00001eb426690*	Hexosyltransferase
ASI	*qASI_DS10.1*	S10_1280961	10	1,280,961	*Zm00001d023240*	Serine/threonine-protein kinase BLUS1
EPO	*qEPO_DS3.3*	S3_230335236	3	230,335,236	*Zm00001d044508*	Cysteine-rich receptor-like protein kinase 2
EPO	*qEPO_DS4.1*	S4_157554490	4	157,554,490	*Zm00001d051412*	CGI-like protein
EPO	*qEPO_DS10.1*	S10_132009667	10	132,009,667	*Zm00001eb426230*	GDSL-motif esterase/acyltransferase/lipase
EPO	*qEPO_DS10.3*	S10_145943876	10	145,943,876	*Zm00001d026371*	anthocyanidin reductase3
EPP	*qEPP_DS1.2*	S1_79999858	1	79,999,858	*Zm00001eb021070*	chloroplast thymidine kinase 1
EPP	*qEPP_DS2.1*	S2_8915850	2	8,915,850	*Zm00001d002247*	MATH-BTB domain proteins
EPP	*qEPP_DS10.1*	S10_138487351	10	138,487,351	*Zm00001eb428440*	TNF receptor-associated factor 38
**Optimum conditions**						
GY	qGY_OPT2.1	S2_188371742	2	188,371,742	*Zm00001d005785*	pentatricopeptide (PPR) repeat-containing protein
GY	qGY_OPT1.1	S1_191855978	1	191,855,978	*Zm00001eb034820*	PfkB-like carbohydrate kinase family protein
GY	qGY_OPT10.1	S10_128990374	10	128,990,374	*Zm00001eb425230*	Trihelix-transcription factor 25
AD	qAD_OPT1.1	S1_287728649	1	287,728,649	*Zm00001d034188*	cold-regulated 413-plasma membrane 2
SD	qSD_OPT1.1	S1_84434846	1	84,434,846	*Zm00001eb022070*	ABI3VP1 transcription factor
ASI	qASI_OPT6.2	S6_166798723	6	166,798,723	*Zm00001d038908*	Lipid phosphate phosphatase 2
PH	qPH_OPT1.2	S1_242503021	1	242,503,021	*Zm00001eb047160*	ks1—kaurene synthase1
PH	qPH_OPT1.3	S1_283188006	1	283,188,006	*Zm00001eb057310*	actin depolymerizing factor 15
PH	qPH_OPT2.2	S2_2224782	2	2,224,782	*Zm00001d001877*	Phytosulfokine receptor 1
PH	qPH_OPT6.1	S6_96553422	6	96,553,422	*Zm00001eb274400*	ALA-interacting subunit 1
EH	qEH_OPT1.1	S1_7555731	1	7,555,731	*Zm00001eb002720*	4-coumarate–CoA ligase
EH	qEH_OPT1.2	S1_86945492	1	86,945,492	*Zm00001eb022630*	NEP1-interacting protein-like 1
EH	qEH_OPT10.1	S10_141529588	10	141,529,588	*Zm00001eb429480*	gar2—gibberellin responsive2
EH	qEH_OPT10.2	S10_144933522	10	144,933,522	*Zm00001eb431080*	bhlh7—bHLH-transcription factor 7
EH	qEH_OPT10.3	S10_88758826	10	88,758,826	*Zm00001eb417450*	HXXXD-type acyl-transferase family protein
EH	qEH_OPT2.1	S2_2224782	2	2,224,782	*Zm00001d001877*	Phytosulfokine receptor 1
EH	qEH_OPT3.1	S3_221276451	3	221,276,451	*Zm00001d044181*	S-type anion channel SLAH3
EH	qEH_OPT5.1	S5_3429314	5	3,429,314	*Zm00001d013023*	Protein GLE1
EH	qEH_OPT8.1	S8_8273703	8	8,273,703	*Zm00001d008420*	bHLH-transcription factor 203
EPO	qEPO_OPT3.1	S3_223539203	3	223,539,203	*Zm00001d044273*	stomatal cytokinesis defective
EPO	qEPO_OPT10.1	S10_106248373	10	106,248,373	*Zm00001eb420220*	Calmodulin-binding domain-containing protein
EPO	qEPO_OPT10.2	S10_144933522	10	144,933,522	*Zm00001eb431080*	BHLH transcription factor (Transcription factor PIF4)
EPP	qEPP_OPT10.2	S10_14445038	10	14,445,038	*Zm00001d023664*	ABA-responsive protein
EPP	qEPP_OPT1.1	S1_8623324	1	8,623,324	*Zm00001eb003210*	CO-LIKE TIMING OF CAB1 protein domain101
EPP	qEPP_OPT10.1	S10_10170518	10	10,170,518	*Zm00001eb408080*	TCP-transcription factor 12

*¥ GY* Grain yield, *AD* days to 50% anthesis, *SD* Days to 50% silking, *ASI* Anthesis-silking interval, *EPH* Ear position (Ear–plant height ratio), *EPP* Ear per plant, *PH* Plant height, *EH* Ear height, *€ Chrom* Chromosome

### Haplotype analysis

**Fig. 6:**
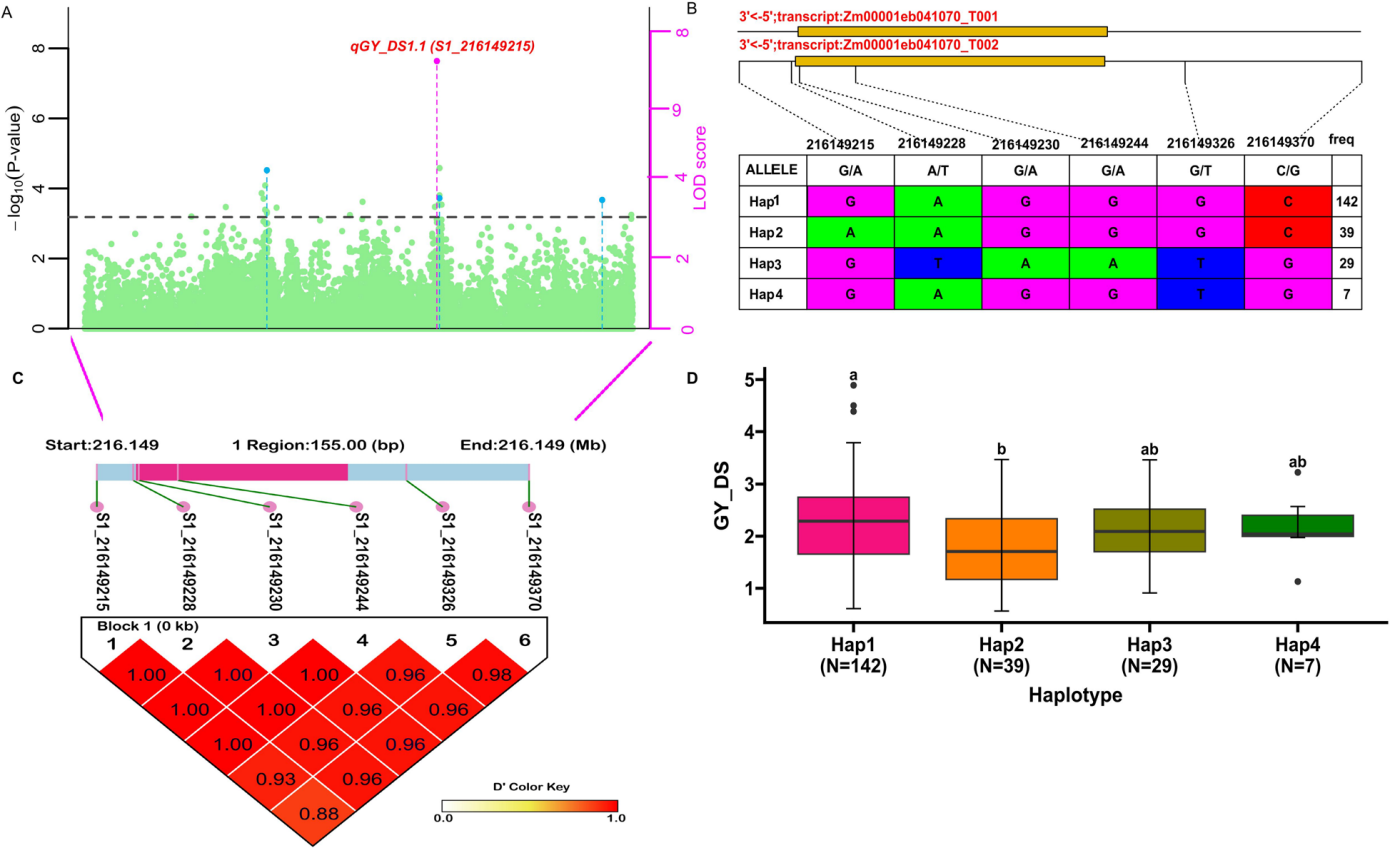
Regional Manhattan plot, haplotype block analysis of *qGY_DS1.1 (S1_216149215)*, and haplotype effect for GY under drought (**A**). Regional Manhattan plot of peak QTN, *qGY_DS1.1 (S1_216149215)* on chromosome 1. Y-axis on the left indicates –log10 (P-values) of QNT, Y-axis on the right indicates the LOD scores. The dashed line represents LOD score of 3. The pink point indicates peak QTNs simultaneously detected indicates least two models (**B**). Gene structure of *Zm00001ed41070* related to peak *qGY_DS1.1 (S1_216149215)* on chr1 and haplotype of *qGY_DS1.1 (S1_216149215)* from 217 maize line (**C**). LD block of peak QTN, *qGY_DS1.1 (S1_216149215)* of region of 155 bp nearest to candidate gene, *Zm00001ed41070.* The thick sky blue and pinks segments represent a strand of a chromosome with QTN/SNP positions*.* The high intensity of the red blocks indicate the D prime value are close to one indicating the six QTNs/SNP are in very high linkage disequilibrium or QTN/SNPs inherited together. **D** Boxplot and phenotypic difference between four haplotypes based on BLUP value of GY using Tukey’s HSD test. Haplotypes with the same letters are not significantly different. N is the number of lines grouped in the same haplotype group

The QTN *qGY_DS1.1* (*S1_216149215*) associated with GY under DS was identified in a 216.15 Mb region on chromosome 1 based on pairwise LD correlation (Fig. 6A and 6C). The QTN region contains six distinct SNPs with relatively high LD (Fig. 6C). Four major haplotypes were detected among the 236 lines with Haplotypes frequencies of 142 (60%), 39 (17%), 29 (12%), and 7 (3%) for Hap1 (GAGGGC), Hap2 (AAGGGC), Hap3 (GTAATG), and Hap4 (GAGGTG), respectively (Fig. 6B). A significant difference was observed between haplotypes Hap1 and Hap2 (Fig. 6D). The mean GY was 2.2 t/ha for Hap1, 1.80 t/ha for Hap2, 2.10 t/ha for Hap3, and 2.2 t/ha for Hap4, respectively. (Fig. 6D). Hap1 was considered a superior haplotype since it contributes to the highest mean performance compared to other haplotypes.

### Genomic prediction

**Fig. 7:**
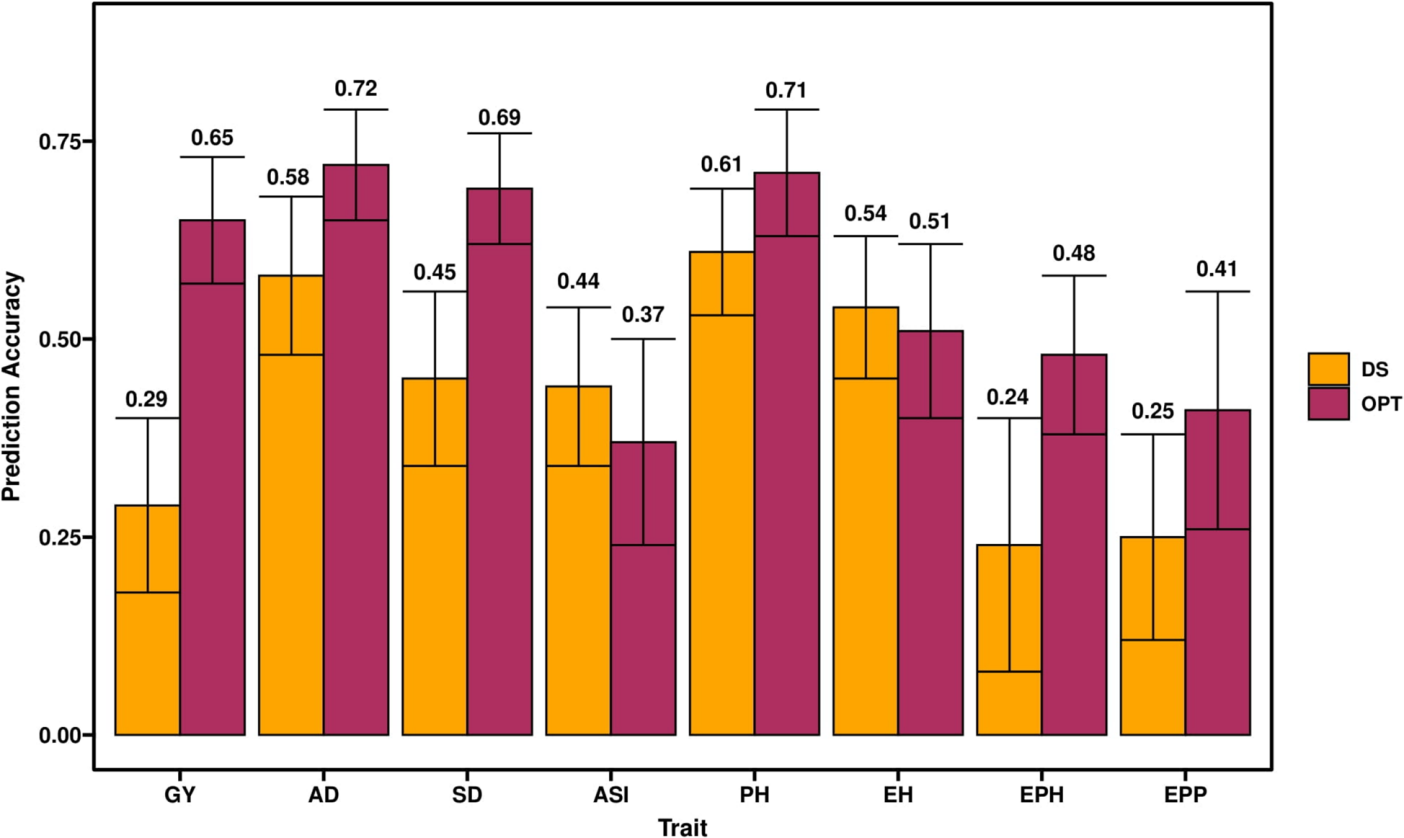
Genomic prediction accuracies for grain yield (GY) and other agronomic traits evaluated under optimum and drought conditions

Among the GP models, RR-BLUP is computationally less intensive and is well suited for routine application in plant breeding trials. Therefore, we used the RR-BLUP model to estimate the performance of maize genotypes for various traits under optimum and DS conditions (Fig. 7). Prediction accuracies were moderate to high for all eight traits under optimum and low to moderate under DS conditions (Fig. 7). The observed prediction accuracy for GY, AD, SD, ASI, PH, EH, EPO, and EPP were 0.29, 0.58, 0.45, 0.44, 0.61, 0.54, 0.24, and 0.25, respectively under DS conditions, and 0.65, 0.72, 0.69, 0.37, 0.71, 0.51, 0.48, and 0.41, under optimum conditions.

## Discussion

Drought has long been recognized as a major abiotic factor limiting crop growth and productivity [4, 10, 87]. Genetic dissection of the genomic regions responsible for GY and other secondary traits under DS will allow breeders to improve their breeding efficiency in the development of climate-resilient varieties and, also, facilitate the introgression of the favorable alleles into elite germplasm using marker-assisted selection [88]. Furthermore, understanding the complex genetic basis of drought tolerance, GY and other secondary traits facilitate the opportunity to test for the indirect selection of GY under DS [25, 89].

In the present study, significant genotypic variance and wide range phenotypic variation observed for all traits under optimal and DS, indicated the presence of adequate genetic diversity within the GWAS panel and that progress from selection for GY and secondary traits could be achieved through breeding. These findings support earlier studies [7, 90, 91], which indicated the existence of sufficient genetic variability. The notable significant GEI in the study implied that the testing sites, drought and optimal growing conditions were discriminating enough in identifying genotypic differences in the response of the GWAS panel to drought and optimal conditions, and that these differences can be largely attributed to the differences in environmental factors, such as soil types, temperature, and amount of rainfall [13]. Heritability estimates are crucial for ascertaining the effectiveness and progress anticipated from future phenotypic selection for yield and secondary traits under DS. The lower heritability estimates for GY under drought stress indicated that the correlated secondary trait, ASI with high heritability and strong correlation with GY could enhance the effectiveness of the selection response [92]. These findings are consistent with earlier studies [90, 91, 93, 94]. A study by Ndlovu et al. [95] on multiple bi-parental maize populations in Kenya under water-stressed and well-watered conditions also reported lower heritability for GY and low genotypic variance under DS conditions. The notably significant and negative correlation between GY and ASI under DS indicated that ASI is a suitable secondary trait to facilitate the selection of GY and a target for improving drought tolerance in maize. These findings are consistent with earlier studies [94, 96, 97].

### Linkage disequilibrium, population structure and association mapping

The LD decay distance determines the power of the GWAS [98]. The high-resolution provided by GWAS is largely influenced by the nature of LD and the extent of its decay across the genome [29, 30], which is population specific and influenced by recombination rate, number of generations of recombination, genetic drift, selection within populations, and population admixture [75, 99]. In this study, genome-wide LD analysis revealed that the GWAS panel decayed rapidly at 4.75 kb (r^2^ = 0.2). This suggests that the GWAS panel contained adequate genetic diversity suitable for GWAS analysis resulting from the historical and evolutionary recombination events present in the panel. Previous studies conducted by Rashid et al.[100], reported a rapid LD decay of 0.9 kb at r^2^ = 0.2 for CIMMYT Asia Association Mapping, 1.75 kb at r^2^ = 0.2 for Drought Tolerant Maize for Africa (DTMA) and 0.99 kb at r^2^ = 0.2 for Improved Maize for African Soils (IMAS) panels. Lu et al. [101], reported that LD decay distance in temperate germplasm (10 to 100 kb), was two to ten times greater than LD decay distance in tropical germplasm (5 to 10 kb), suggesting that tropical maize has significant genetic diversity for breeding programs. The population structure within the GWAS panel was best explained when separated into four groups. The incorporation of the four PCs in GWAS analysis was sufficient to reduce false positive marker-trait association in the present study as evident in the Q-Q plots and Manhattan plots.

The variation in the number of QTNs detected across the GWAS models suggests that the differences can be attributed to genetic algorithms implemented in the different models. A similar conclusion that multi-locus models show superior performance over single-locus models in terms of statistical power in detecting QTNs, particularly in the accuracy of QTN effect estimation and reducing false positive rates has been found in many other studies [33, 41, 102, 103]. This further implied that adopting multiple GWAS models could help to complement each other in identifying reliable and stable QTNs [7]. The proportion of phenotypic variance explained R^2^ (%) by each QTNs and the QTN effects observed for GY, EPP and EPO in this study were relatively low under DS. The results implied that the GY, EPP and EPO under DS are likely influenced by numerous QTNs with small effect. Similar results were reported in earlier study [104].

### Candidate genes discovery

In the present study, two candidate genes (*Zm00001eb041070 and Zm00001d045665*) significantly associated with GY were identified under DS. The peak QTNs, *qGY_DS1.1* is located within the gene, *Zm00001eb041070* encoding AP2 (*APETALA2*)-*EREBP* (Ethylene Responsive Element Binding Protein) transcription factor 60 on chromosome 1. It plays a critical role in regulating genes involved in plant growth and development, biotic, and abiotic stress [105]. Ningning et al. [106] reported three candidate genes, *GRMZM2G322672 (EREB37), GRMZM2G026926 (ERF), and GRMZM2G169654 (RAV)*, belonging to the *AP2/EREBP* family that plays important roles in maize response to DS. In maize, *ZmEREBP60* is a positive regulator under DS and its expression, significantly upregulated in the roots, coleoptiles, and leaves in response to drought [107, 108] Another gene *Zm00001eb041070* reported to control AD under DS [104, 109]. In addition, a gene *Zm00001d045665* encoding Ubiquitin receptor Radiation sensitivity proteins-23 (RAD23) was identified -46 bp around the peak QTN, *qGY_DS.9.1*. This receptor is known to participate in DNA repair and play a major role in the cell cycle, morphology, and fertility of plants through their delivery of UPS substrates to the 26S proteasome [110, 111].

The QTNs associated with GY, *qGY_OPT2.1*, *qGY_OPT1.1*, *qGY_OPT10.1* are nearest to three candidate genes, *Zm00001d005785, Zm00001eb034820 and Zm00001eb425230* which encode pentatricopeptide (PPR) repeat-containing protein [112, 113], PfkB-like carbohydrate kinase family protein[114] and Trihelix-transcription factor (TTF) 25 [115, 116], respectively. In a QTN-by environment interaction GWAS under optimum, DS, heat stress, and combined drought and heat stress, Wen et al. [104] identified *GRMZM2G110851* related to GY around the locus *S1_299093763* encoding pentatricopeptide repeat protein, required for mitochondrial function and kernel development in maize. In rice and Arabidopsis, phosphofructokinase B-type carbohydrate kinase family protein, PFKB1 regulates leaf and plant growth by controlling chloroplast biogenesis[114, 117]. The TTFs are light-responsive proteins and play a critical role in plant growth and development and stress tolerance in maize [116, 118].

Flowering traits in maize are quantitative in nature and regulated by complex genetic mechanisms [119]. Several candidate genes associated with flowering traits under DS and optimal conditions have been reported in maize[24, 54, 104, 120, 121]. Here, some new candidates have been found to be associated with flowering traits. Rubisco large subunit methyltransferase encoded by *Zm00001eb058200* on chromosome 1 near peak QTN *qAD_DS1.1* was found under DS. It is a key photosynthetic enzyme in the chloroplast that catalyzes the fixation of atmospheric CO_2_ during photosynthesis in the carbon assimilation pathway and drought-related responses [122, 123]. The gene *Zm00001eb010210*, associated with AD under DS encodes plant U-box type E3 ubiquitin ligase [124, 125]. The ligase gene family plays a key role in the abiotic stress adaptation in maize [126]. Another candidate gene for AD, *Zm00001eb012590* encodes Trifunctional UDP-glucose 46-dehydratase, an enzyme plays a key role in nucleotide sugar biosynthetic pathway, maintaining cell wall integrity during maize cell growth and response to abiotic stress[127, 128].

ASI is a key index in breeding programs for facilitating indirect selection for GY under DS. Maize exhibits postponement in the silking process under stress, resulting in an extended period between AD and SD [89]. Five candidate genes *Zm00001eb047160, Zm00001eb364110, Zm00001eb430770, Zm00001eb426690 and Zm00001d023240* associated with ASI under DS, encodes for kaurene synthase1[129], *TEOSINTE BRANCHED1/CYCLOIDEA/PROLIFERATING CELL FACTOR (TCP)* -transcription factor 20 [130], Ribosomal protein s11-beta [131], Hexosyltransferase[132] and Serine/threonine-protein kinase BLUS1[133], respectively. Additionally, the candidate gene, *Zm00001eb047160* associated with PH under optimal conditions, encodes kaurene synthase, an enzyme responsible for the biosynthesis of gibberellins and phytoalexin metabolism [129, 134]. The TCP family of transcription factors participates in the regulation of cell growth, proliferation, and response to abiotic stress [135]. In maize, *ZmGA20ox3* play a crucial in regulating PH and enhancing drought tolerance [136]. Furthermore, candidate genes, *Zm00001eb364110* and *Zm00001eb408080* related to ASI and EPP on chromosome 8 and 10, respectively, under drought and optimal conditions encode TCP-transcription factor [137, 138]. Previous studies reported that *ZmTCP* family proteins, particularly *ZmTCP42*, is a key positive regulator of drought tolerance in maize. However, overexpression of the *ZmTCP14 gene* significantly reduced the tolerance to drought [138]. However, *ZmTCP14* gene-edited plants showed improved drought tolerance and GY[137, 138]. Additionally, the candidate gene *Zm00001d023240* encodes serine/threonine-protein kinase, *BLUE LIGHT SIGNALLING 1 (BLUS1)*, play a crucial role in blue light-induced stomatal opening and transduction of signals related to abiotic stress [137, 139]. This suggests that serine/threonine-protein kinase BLUS1 participates in regulating ASI during DS.

Among the candidate genes identified with association to EPO under DS, *Zm00001d044508* encodes cysteine-rich receptor kinase, which plays a critical role in plant immunity, and abiotic stress response [113, 114]. Another gene *Zm00001eb426230* encodes GDSL-motif esterase/acyltransferase/lipase*,* play a key role in cuticle development, seed oil storage, and biotic and abiotic stress responses [140–142]. The *Zm00001d026371* gene associated with EPO encodes anthocyanidin reductase, a key enzyme in involved in mediating proanthocyanidin and lignin biosynthesis in response to DS [143, 144]. Previous study [145] reported that the anthocyanidin reductase enzyme system plays a key role in reducing excess reactive oxygen species (ROS) induced by DS, which can damage cellular membranes and cause cell death in maize. The *Zm00001d002247* gene, which underpinned an association with EPP under DS, encodes the MATH-BTB domain containing proteins, which play a key role in abiotic stress response [146, 147].

The candidate gene *Zm00001d001877* near peak QTN, *qEH_OPT2.1* and *qPH_OPT2.2* exhibited pleiotropic effect on EH and PH under optimal conditions. This implied that a positive correlation between these two traits and that the two QTNs simultaneously regulated [148]. The candidate gene, *Zm00001eb431080* on chromosome 10 exhibited pleiotropic effect with PH and EPO under optimum conditions. This candidate gene encodes basic Helix-Loop-Helix (*bHLH*) transcription factor, involved in anthocyanin biosynthesis and response to biotic and abiotic stresses [149–151]. ABA-responsive protein encoded by *Zm00001d023664* is associated with EPP plays a critical role in controlling stomatal closure, and regulate plants response to abiotic stress [152, 153]. The candidate gene, *Zm00001eb003210* associated with EPP encodes *CONSTANS-LIKE (COL) TIMING OF CAB1* protein domain, which plays a crucial role in regulating flowering through photoperiodic control and response to abiotic stress [154, 155].

### Haplotype estimation

Haplotype refers to a set of alleles combinations within a specific genomic region showing significant LD and are inherited together [83, 156]. Identification of superior haplotypes linked to functional genes is important for developing markers to accelerate genetic gain and facilitate efficient selection in breeding operations. The haplo-pheno analysis identified Hap1 (GAGGGC) as a superior haplotype with high mean performance associated GY near QTN, *qGY_DS1.1* (*S1_216149215*) in the present study. Incorporating this superior haplotype in maize breeding will facilitate the selection of improved drought tolerant maize cultivars. These findings revealed that the GY of lines carrying superior haplotypes can exhibit improved drought tolerance compared to lines with unfavorable haplotypes. This is evident in their ability to maintain higher yields in DS and, these lines can also be utilized as parents for hybridization and backcrossing programs in the future.

### Genome-wide prediction

GS has been successfully applied in maize for complex traits like GY under optimum, low soil N stress, heat stress and DS conditions [7, 55, 57, 157]. In the present study, we compared the prediction accuracies under optimum and DS conditions (Fig. 7). The prediction accuracies were moderate to high for GY and other traits under optimum conditions whereas the accuracies were slightly lower under DS conditions. The accuracy observed for all traits under both optimum and DS conditions reveal the effect of heritability as the traits with higher heritability generally had higher prediction accuracy. The observed prediction accuracies for GY and other traits are comparable to earlier studies reported under different stresses in maize [5, 7, 57, 95, 157, 158]. In genome-wide predictions, the less complex traits like AD, SD and PH had higher accuracy compared to the GY, which is consistent with the nature of trait complexity [7, 55, 56].

Breeding for drought tolerance is complex and very expensive. The observed prediction accuracy for GY is 0.29 and 0.65 under DS and optimum conditions, respectively. On the other hand, the phenotypic selection efficiency is 0.50 (square root of heritability of GY) under DS and 0.73 under optimum conditions. Nevertheless, with the possibility to complete three cycles per year and requirement of lesser resources for implementing GS compared to phenotypic selection, endorse GS to integrate to improve the selection efficiency for drought tolerance as an attractive option in a longer run objective of breeding program. Further, integration of GS with GWAS results leads not only to an increase in the prediction accuracy, but also helps to validate the function of the identified candidate genes and increases in the accumulation of favorable alleles for drought tolerance in improved set of elite lines. Further GS can remarkably reduce the resources required for selection and improve breeding efficiency.

## Conclusion

Tremendous variations for GY and secondary traits associated with DS existed in the maize GWAS panel used in this study. Based on seven GWAS models, a total of 172 stable and reliable QTNs (77 under DS and 95 under optimal conditions) were identified. Among these QTNs two QTNs, *qGY_DS1.1* for GY and *qASI_DS8.2* for ASI were novel. This result demonstrated that combining different GWAS models is effective and powerful in exploiting the complementary strengths of different methods in identifying QTNs with small and large effects for GY and secondary traits with a complex genetic basis and low heritability under DS. In addition, a total of 43 candidate genes were detected (18 under DS and 25 optimum conditions). Among these, two key candidate genes are closely associated with GY and nine closely associated with flowering traits under DS. Genomic prediction revealed moderate to high accuracies under optimum and DS conditions. Integration of GS with GWAS results leads not only to an increase in prediction accuracy, but also helps to increase in the accumulation of favorable alleles for drought tolerance. Overall stable QTNs and candidate genes for GY and secondary trait detected in the current study can serve as invaluable resources for improving GY under DS in maize. The identified superior haplotype can be converted to a breeder-friendly marker to increase its favorable alleles in elite breeding lines. These findings provide valuable insight into the genetic basis of drought tolerance for GY and secondary traits in tropical maize.

## Supplementary Information

Supplementary Material 1: Supplementary Figure S1. Population structure analyses of 236 maize inbred lines based on 215,542 SNPs : (a) Evanno plot of the number of clusters (K) against delta K to determine the optimum number of K; (b) graphical representation of the 236 lines at K = 3 to K = 10. Each individual is shown as a vertical line divided into K colored segments, with segment lengths indicating the estimated probability of membership to each cluster.

Supplementary Material 2: Supplementary Figure S2. Farm CPU-based Manhattan and Q-Q plots of genome-wide association study (GWAS) on eight traits evaluated under optimum (_OPT) and drought (_DS) environmental conditions. The − log10(*p*) values on the Y-axis in the Manhattan plot represent grain yield (GY), Days to 50 % anthesis, (AD), Days to 50 % silking (SD), Anthesis-Silking Interval (ASI), Plant height (PH), Ear height (EH), Ear position (EPO) and Ear per plant (EPP) plotted against chromosome position on X-axis. The red and blue solid horizontal lines in the Manhattan plots represent the genome-wide (− log10 (p) =6.2). The quantile—quantile plots represent observed against the expected −log10 (*p*).

Supplementary Material 3: Supplementary Figure S3. Manhattan and Q-Q plots of genome-wide association study (GWAS) on eight traits evaluated under optimum (OPT) and drought (_DS) environmental conditions for six GWAS models. The pink dots above the threshold indicates significant QTNs identified by more than one ML-GWAS models, while green and blue dots above the threshold represent significant QTNs identified by a single ML-GWAS model . The black horizontal dashed line indicates the genome-wide significance threshold, corresponding to a −log10 (*p*) value of a LOD score ≥ 3.0 for ML-GWAS models.

Supplementary Material 4: Supplementary Table S1. Detailed information about pedigree of 236 lines and groupings based on markers used in the study. Supplementary Table S2. Summary of SNPs distribution across the ten maize chromosomes. Supplementary Table S3. Significant QTNs identified for eight traits across multi-environment trials under optimum conditions using six multi-locus GWAS models. Supplementary Table S4. Significant QTNs identified for eight traits across multi-environment trials under drought conditions using six multi-locus GWAS models. Supplementary Table S5. Significant QTNs associated with eight traits under drought and optimum condition using Farm CPU GWAS model from GAPIT. Supplementary Table S6. Number of stable QTNs detected by at least two GWAS models for grain yield, flowering traits and other agronomic traits under drought and optimum conditions.

## Data Availability

The original data presented in the study are included in the supplementary materials (phenotypic data) and are accessible via https://hdl.handle.net/11529/10549140 (genotypic data). Further inquiries can be directed to the corresponding author.

## Abbreviations

ANOVA: Analysis of variance
AD: Anthesis days
ASI: Anthesis-silking interval
BLINK: Bayesian-information and linkage-disequilibrium iteratively nested keyway
CV: Coefficient of variation
CIMMYT: International Maize and Wheat Improvement Centre
DS: Drought Stress
ECMLM: Enriched compressed mixed linear model
EMMAX: Efficient mixed model association eXpedited
EPO: Ear position
EH: Ear height
EPP: Ear per plant
FarmCPU: Fixed and random model Circulating Probability Unification
FaST-LMM: Factored spectrally transformed linear mixed models
FASTmrEMMA: Fast multi-locus random-SNP-effect efficient mixed model analysis
FASTmrMLM: Fast mrMLM
GP: Genomic prediction
GS: Genomic selection
GY: Grain yield
GLM: General linear model
GWAS: Genome-wide association study
GEMMA: Genome-wide efficient mixed-model association
ISIS EM-BLASSO: Iterative modified-sure independence screening expectation–maximization-Bayesian LASSO
LD: Linkage disequilibrium
LOD: Logarithm of Odds
ML-GWAS: Multi-locus genome-wide association studies
MLM: Mixed linear model
mrMLM: Multi-locus random-SNP-effect MLM
PCA: Principal component analysis
pLARmEB: Polygenic-background-control-based least angle regression plus empirical Bayes
pKWmEBb: Integration of Kruskal–Wallis test with empirical Bayes
QTL: Quantitative trait locus
QTN: Quantitative trait nucleotide
R^2^: The proportion of total phenotypic variance explained by each QTN
r^2^: The squared correlation coefficient
SL-GWAS: Single-locus genome-wide association studies
SNP: Single-nucleotide polymorphic

## References

[1] Erenstein O, Jaleta M, Sonder K, Mottaleb K, Prasanna BM. Global maize production, consumption and trade: Trends and R&D implications. Food Secur. 2022;14:1295–319.

[2] FAO. The Global Action for Fall Armyworm Control: Action framework 2020–2022. Working together to tame the global threat. Rome: 2020.

[3] Prasanna BM, Nair SK, Babu R, Gowda M, Zhang X, Xu Y, et al. Increasing genetic gains in maize in stress-prone environments of the tropics. In: Kole C, editor. Genomic Designing of Climate-Smart Cereal Crops. Cham: Springer International Publishing; 2020. p. 97–132.

[4] Tarekegne A, Wegary D, Cairns JE, Zaman-Allah M, Beyene Y, Negera D, et al. Genetic gains in early maturing maize hybrids developed by the International Maize and Wheat Improvement Center in Southern Africa during 2000–2018. Front Plant Sci. 2023;14:1321308. 38293626 10.3389/fpls.2023.1321308 PMC10825029

[5] Ndlovu N, Spillane C, McKeown PC, Cairns JE, Das B, Gowda M. Genome-wide association studies of grain yield and quality traits under optimum and low-nitrogen stress in tropical maize (Zea mays L.). Theor Appl Genet. 2022;135:4351–70. 10.1007/s00122-022-04224-7 PMC9734216 36131140

[6] Raza A, Mubarik MS, Sharif R, Habib M, Jabeen W, Zhang C, et al. Developing drought-smart, ready-to-grow future crops. Plant Genome. 2023;16:e20279. 36366733 10.1002/tpg2.20279 PMC12807413

[7] Yuan Y, Cairns JE, Babu R, Gowda M, Makumbi D, Magorokosho C, et al. Genome-wide association mapping and genomic prediction analyses reveal the genetic architecture of grain yield and flowering time under drought and heat stress conditions in maize. Front Plant Sci. 2019;9:1–15. 10.3389/fpls.2018.01919 PMC6363715 30761177

[8] Masuka B, Magorokosho C, Olsen M, Atlin GN, Bänziger M, Pixley K V., et al. Gains in maize genetic improvement in eastern and southern Africa: II. CIMMYT open-pollinated variety breeding pipeline. Crop Sci. 2017;57:180–91.

[9] Fisher M, Abate T, Lunduka RW, Asnake W, Alemayehu Y, Madulu RB. Drought tolerant maize for farmer adaptation to drought in sub-Saharan Africa: Determinants of adoption in eastern and southern Africa. Clim Change. 2015;133:283–99.

[10] Prasanna BM, Cairns JE, Zaidi PH, Beyene Y, Makumbi D, Gowda M, et al. Beat the stress: breeding for climate resilience in maize for the tropical rainfed environments. Theor Appl Genet. 2021;134:1729–52. 33594449 10.1007/s00122-021-03773-7 PMC7885763

[11] Brouwer C and Heibloem, M. Irrigation Water Management: Irrigation Water Needs. Training Manual 3, FAO. Rome: 1986.

[12] He Z, Zhang T, Liu X, Shang X. Water-yield relationship responses of maize to ridge-furrow planting systems coupled with multiple irrigation levels in china’s horqin sandy land. Agronomy. 2018;8:221.

[13] Badu-Apraku B, Fakorede MAB. Breeding Maize for Drought Tolerance. In: Advances in Genetic Enhancement of Early and Extra-Early Maize for Sub-Saharan Africa. Cham: Springer International Publishing; 2017. p. 287–309.

[14] Menkir A, Crossa J, Meseka S, Bossey B, Muhyideen O, Riberio PF, et al. Stacking Tolerance to Drought and Resistance to a Parasitic Weed in Tropical Hybrid Maize for Enhancing Resilience to Stress Combinations. Front Plant Sci. 2020;11:166. 32194590 10.3389/fpls.2020.00166 PMC7061855

[15] Ao S, Russelle MP, Varga T, Feyereisen GW, Coulter JA. Drought tolerance in maize is influenced by timing of drought stress initiation. Crop Sci. 2020;60:1591–606.

[16] Edmeades GO, Trevisan W, Prasanna BM, Campos H. Tropical Maize (Zea mays L.). In: Genetic Improvement of Tropical Crops. Cham: Springer International Publishing; 2017. p. 57–109.

[17] Menkir A, Dieng I, Gedil M, Mengesha W, Oyekunle M, Riberio PF, et al. Approaches and progress in breeding drought‐tolerant maize hybrids for tropical lowlands in west and central Africa. The Plant Genome. 2024;17:e20437. 10.1002/tpg2.20437 PMC12806867 38379199

[18] McMillen MS, Mahama AA, Sibiya J, Lübberstedt T, Suza WP. Improving drought tolerance in maize: Tools and techniques. Front Genet. 2022;13:1–13. 10.3389/fgene.2022.1001001 PMC9651916 36386797

[19] Bänziger M, Edmeades GO, Beck D, Bello M. Breeding for drought and nitrogen stress tolerance in maize: from theory to practice. Mexico: CIMMYT; 2000.

[20] Cooper M, Gho C, Leafgren R, Tang T, Messina C. Breeding drought-tolerant maize hybrids for the US corn-belt: Discovery to product. J Exp Bot. 2014;65:6191–4. 24596174 10.1093/jxb/eru064

[21] Cooper M, Messina CD. Breeding crops for drought-Affected environments and improved climate resilience. Plant Cell. 2023;35:162–86. 36370076 10.1093/plcell/koac321 PMC9806606

[22] Wang N, Liu B, Liang X, Zhou Y, Song J, Yang J, et al. Genome-wide association study and genomic prediction analyses of drought stress tolerance in China in a collection of off-PVP maize inbred lines. Mol Breeding. 2019;39:113.

[23] Varshney RK, Barmukh R, Bentley A, Nguyen HT. Exploring the genomics of abiotic stress tolerance and crop resilience to climate change. Plant Genome. 2024:1–6. 10.1002/tpg2.20445 PMC12806857 38481118

[24] Li C, Sun B, Li Y, Liu C, Wu X, Zhang D, et al. Numerous genetic loci identified for drought tolerance in the maize nested association mapping populations. BMC Genomics. 2016;17:1–11. 27825295 10.1186/s12864-016-3170-8 PMC5101730

[25] Liu S, Qin F. Genetic dissection of maize drought tolerance for trait improvement. Mol Breeding. 2021;41:8. 10.1007/s11032-020-01194-w PMC10236036 37309476

[26] Shi W, Hao C, Zhang Y, Cheng J, Zhang Z, Liu J, et al. A combined association mapping and linkage analysis of kernel number per spike in common wheat (Triticum aestivum L.). Front Plant Sci. 2017;8:1–13. 28868056 10.3389/fpls.2017.01412 PMC5563363

[27] Davoud Torkamaneh FB. Genome-Wide Association Studies. 1st edition. New York, NY: Humana New York, NY; 2022.

[28] Alseekh S, Kostova D, Bulut M, Fernie AR. Genome-wide association studies: assessing trait characteristics in model and crop plants. Cell Mol Life Sci. 2021;78:5743–54. 34196733 10.1007/s00018-021-03868-w PMC8316211

[29] Xiao Y, Liu H, Wu L, Warburton M, Yan J. Genome-wide Association Studies in Maize: Praise and Stargaze. Mol Plant. 2017;10:359–74. 28039028 10.1016/j.molp.2016.12.008

[30] Tibbs Cortes L, Zhang Z, Yu J. Status and prospects of genome‐wide association studies in plants. The Plant Genome. 2021;14:e20077. 10.1002/tpg2.20077 PMC12806871 33442955

[31] Hao D, Xue L, Zhang Z, Cheng Y, Chen G, Zhou G, et al. Combined linkage and association mapping reveal candidate loci for kernel size and weight in maize. Breed Sci. 2019;69:420–8. 31598074 10.1270/jsbbs.18185 PMC6776153

[32] Zheng H, Chen J, Mu C, Makumbi D, Xu Y, Mahuku G. Combined linkage and association mapping reveal QTL for host plant resistance to common rust (Puccinia sorghi) in tropical maize. BMC Plant Biol. 2018;18:1–14. 30497411 10.1186/s12870-018-1520-1 PMC6267831

[33] An Y, Chen L, Li YX, Li C, Shi Y, Zhang D, et al. Genome-wide association studies and whole-genome prediction reveal the genetic architecture of KRN in maize. BMC Plant Biol. 2020;20:1–11. 33109077 10.1186/s12870-020-02676-x PMC7590725

[34] Price AL, Patterson NJ, Plenge RM, Weinblatt ME, Shadick NA, Reich D. Principal components analysis corrects for stratification in genome-wide association studies. Nat Genet. 2006;38:904–9. 16862161 10.1038/ng1847

[35] Zhang Z, Ersoz E, Lai CQ, Todhunter RJ, Tiwari HK, Gore MA, et al. Mixed linear model approach adapted for genome-wide association studies. Nat Genet. 2010;42:355–60. 20208535 10.1038/ng.546 PMC2931336

[36] Li M, Liu X, Bradbury P, Yu J, Zhang YM, Todhunter RJ, et al. Enrichment of statistical power for genome-wide association studies. BMC Biol. 2014;12:1. 25322753 10.1186/s12915-014-0073-5 PMC4210555

[37] Kang HM, Sul JH, Service SK, Zaitlen NA, Kong SY, Freimer NB, et al. Variance component model to account for sample structure in genome-wide association studies. Nat Genet. 2010;42:348–54. 10.1038/ng.548 PMC3092069 20208533

[38] Zhou X, Stephens M. Genome-wide efficient mixed-model analysis for association studies. Nat Genet. 2012;44:821–4. 22706312 10.1038/ng.2310 PMC3386377

[39] Yoosefzadeh-Najafabadi M, Eskandari M, Belzile F, Torkamaneh D. Genome-Wide Association Study Statistical Models: A Review. In: Methods in Molecular Biology. 2022. p. 43–62. 10.1007/978-1-0716-2237-7_4 35641758

[40] Zhang YW, Tamba CL, Wen YJ, Li P, Ren WL, Ni YL, et al. mrMLM v4.0.2: An R Platform for Multi-locus Genome-wide Association Studies. Genomics, Proteomics Bioinforma. 2020;18:481–7. 10.1016/j.gpb.2020.06.006 PMC8242264 33346083

[41] Zhang YM, Jia Z, Dunwell JM. Editorial: The applications of new multi-locus GWAS methodologies in the genetic dissection of complex traits, volume II. Front Plant Sci. 2023;14:1–3. 10.3389/fpls.2023.1340767 PMC10749431 38146269

[42] Liu X, Huang M, Fan B, Buckler ES, Zhang Z. Iterative Usage of Fixed and Random Effect Models for Powerful and Efficient Genome- Wide Association Studies. PLOS Genet. 2016;12:1–24. 10.1371/journal.pgen.1005767 PMC4734661 26828793

[43] Huang M, Liu X, Zhou Y, Summers RM, Zhang Z. BLINK: A package for the next level of genome-wide association studies with both individuals and markers in the millions. Gigascience. 2019;8:1–12. 10.1093/gigascience/giy154 PMC6365300 30535326

[44] Wang SB, Feng JY, Ren WL, Huang B, Zhou L, Wen YJ, et al. Improving power and accuracy of genome-wide association studies via a multi-locus mixed linear model methodology. Sci Rep. 2016;6:19444. 26787347 10.1038/srep19444 PMC4726296

[45] Tamba CL, Zhang YM. A fast mrMLM algorithm for multi-locus genome-wide association studies. bioRxiv. 2018.

[46] Wen YJ, Zhang H, Ni YL, Huang B, Zhang J, Feng JY, et al. Methodological implementation of mixed linear models in multi-locus genome-wide association studies. Brief Bioinform. 2018;19:700–12. 28158525 10.1093/bib/bbw145 PMC6054291

[47] Zhang J, Feng JY, Ni YL, Wen YJ, Niu Y, Tamba CL, et al. PLARmEB: Integration of least angle regression with empirical Bayes for multilocus genome-wide association studies. Heredity (Edinb). 2017;118:517–24. 28295030 10.1038/hdy.2017.8 PMC5436030

[48] Ren WL, Wen YJ, Dunwell JM, Zhang YM. PKWmEB: Integration of Kruskal-Wallis test with empirical Bayes under polygenic background control for multi-locus genome-wide association study. Heredity (Edinb). 2018;120:208–18. 29234158 10.1038/s41437-017-0007-4 PMC5836593

[49] Tamba CL, Ni YL, Zhang YM. Iterative sure independence screening EM-Bayesian LASSO algorithm for multi-locus genome-wide association studies. PLoS Comput Biol. 2017;13:e1005357. 28141824 10.1371/journal.pcbi.1005357 PMC5308866

[50] Khan N, Shazadee H, Cloutier S, You FM. Genomics Assisted Breeding Strategy in Flax. In: You FM, Fofana B, editors. The Flax Genome. Compendium of Plant Genomes. 1st edition. Springer, Cham; 2023. p. 253–72.

[51] Meuwissen THE, Hayes BJ, Goddard ME. Prediction of total genetic value using genome-wide dense marker maps. Genetics. 2001;157:1819–29. 11290733 10.1093/genetics/157.4.1819 PMC1461589

[52] Crossa J, Pérez-Rodríguez P, Cuevas J, Montesinos-López O, Jarquín D, de los Campos G, et al. Genomic Selection in Plant Breeding: Methods, Models, and Perspectives. Trends Plant Sci. 2017;22:961–75. 10.1016/j.tplants.2017.08.011 28965742

[53] Beyene Y, Semagn K, Mugo S, Tarekegne A, Babu R, Meisel B, et al. Genetic gains in grain yield through genomic selection in eight bi-parental maize populations under drought stress. Crop Sci. 2015;55:154–63.

[54] Wallace JG, Zhang X, Beyene Y, Semagn K, Olsen M, Prasanna BM, et al. Genome-wide association for plant height and flowering time across 15 tropical maize populations under managed drought stress and well-watered conditions in Sub-Saharan Africa. Crop Sci. 2016;56:2365–78.

[55] Zhang A, Wang H, Beyene Y, Semagn K, Liu Y, Cao S, et al. Effect of trait heritability, training population size and marker density on genomic prediction accuracy estimation in 22 bi-parental tropical maize populations. Front Plant Sci. 2017;8:1916. 29167677 10.3389/fpls.2017.01916 PMC5683035

[56] Zhang X, Pérez-Rodríguez P, Semagn K, Beyene Y, Babu R, López-Cruz MA, et al. Genomic prediction in biparental tropical maize populations in water-stressed and well-watered environments using low-density and GBS SNPs. Heredity (Edinb). 2015;114:291–9. 25407079 10.1038/hdy.2014.99 PMC4815582

[57] Beyene Y, Gowda M, Pérez-Rodríguez P, Olsen M, Robbins KR, Burgueño J, et al. Application of Genomic Selection at the Early Stage of Breeding Pipeline in Tropical Maize. Front Plant Sci. 2021;12:1265. 10.3389/fpls.2021.685488 PMC8274566 34262585

[58] Masuka B, Araus JL, Das B, Sonder K, Cairns JE. Phenotyping for Abiotic Stress Tolerance in Maize. J Integr Plant Biol. 2012;54:238–49. 22443263 10.1111/j.1744-7909.2012.01118.x

[59] Zaidi PH. Management of drought stress in field phenotyping. Mexico: CIMMYT; 2019.

[60] Alvarado G, Rodríguez FM, Pacheco A, Burgueño J, Crossa J, Vargas M, et al. META-R: A software to analyze data from multi-environment plant breeding trials. Crop J. 2020;8:745–56.

[61] Bates D, Mächler M, Bolker B, Walker S. Fitting Linear Mixed-Effects Models Using lme4. J Stat Soft. 2015;67.

[62] Kresovich S. Quantitative genetics in maize breeding. F Crop Res. 1990;23:78–9.

[63] Semagn K. Leaf tissue sampling and DNA extraction protocols. Methods Mol Biol. 2014;1115:53–67. 24415469 10.1007/978-1-62703-767-9_3

[64] Elshire RJ, Glaubitz JC, Sun Q, Poland JA, Kawamoto K, Buckler ES, et al. A robust, simple genotyping-by-sequencing (GBS) approach for high diversity species. PLoS ONE. 2011;6:19379. 10.1371/journal.pone.0019379 PMC3087801 21573248

[65] Glaubitz JC, Casstevens TM, Lu F, Harriman J, Elshire RJ, Sun Q, et al. TASSEL-GBS: A high capacity genotyping by sequencing analysis pipeline. PLoS One. 2014;9:e90346. 24587335 10.1371/journal.pone.0090346 PMC3938676

[66] Bradbury PJ, Zhang Z, Kroon DE, Casstevens TM, Ramdoss Y, Buckler ES. TASSEL: Software for association mapping of complex traits in diverse samples. Bioinformatics. 2007;23:2633–5. 17586829 10.1093/bioinformatics/btm308

[67] Hubisz MJ, Falush D, Stephens M, Pritchard JK. Inferring weak population structure with the assistance of sample group information. Mol Ecol Resour. 2009;9:1322–32. 21564903 10.1111/j.1755-0998.2009.02591.x PMC3518025

[68] Pritchard JK, Stephens M, Donnelly P. Inference of population structure using multilocus genotype data. Genetics. 2000;155:945–59. 10835412 10.1093/genetics/155.2.945 PMC1461096

[69] Earl DA, vonHoldt BM. STRUCTURE HARVESTER: A website and program for visualizing STRUCTURE output and implementing the Evanno method. Conserv Genet Resour. 2012;4:359–61.

[70] Evanno G, Regnaut S, Goudet J. Detecting the number of clusters of individuals using the software STRUCTURE: A simulation study. Mol Ecol. 2005;14:2611–20. 15969739 10.1111/j.1365-294X.2005.02553.x

[71] Wang J, Zhang Z. GAPIT Version 3: Boosting Power and Accuracy for Genomic Association and Prediction. Genomics Proteomics Bioinforma. 2021;19:629–40. 10.1016/j.gpb.2021.08.005 PMC9121400 34492338

[72] Lipka AE, Tian F, Wang Q, Peiffer J, Li M, Bradbury PJ, et al. GAPIT: Genome association and prediction integrated tool. Bioinformatics. 2012;28:2397–9. 22796960 10.1093/bioinformatics/bts444

[73] VanRaden PM. Efficient methods to compute genomic predictions. J Dairy Sci. 2008;91:4414–23. 18946147 10.3168/jds.2007-0980

[74] Hill WG, Weir BS. Variances and covariances of squared linkage disequilibria in finite populations. Theor Popul Biol. 1988;33:54–78. 3376052 10.1016/0040-5809(88)90004-4

[75] Remington DL, Thornsberry JM, Matsuoka Y, Wilson LM, Whitt SR, Doebley J, et al. Structure of linkage disequilibrium and phenotypic associations in the maize genome. Proc Natl Acad Sci U S A. 2001;98:11479–84. 11562485 10.1073/pnas.201394398 PMC58755

[76] Li MX, Yeung JMY, Cherny SS, Sham PC. Evaluating the effective numbers of independent tests and significant p-value thresholds in commercial genotyping arrays and public imputation reference datasets. Hum Genet. 2012;131:747–56. 22143225 10.1007/s00439-011-1118-2 PMC3325408

[77] Gao X, Starmer ÃJ, Martin ER. A Multiple Testing Correction Method for Genetic Association Studies Using Correlated Single Nucleotide Polymorphisms. Genet Epedemiology. 2008;369:361–9. 10.1002/gepi.20310 18271029

[78] Slaten ML, Chan YO, Shrestha V, Lipka AE, Angelovici R. HAPPI GWAS: Holistic analysis with pre- And post-integration GWAS. Bioinformatics. 2020;36:4655–7. 32579187 10.1093/bioinformatics/btaa589

[79] Jiao Y, Peluso P, Shi J, Liang T, Stitzer MC, Wang B, et al. Improved maize reference genome with single-molecule technologies. Nature. 2017;546:524–7. 10.1038/nature22971 PMC7052699 28605751

[80] Schnable PS, Ware D, Fulton RS, Stein JC, Wei F, Pasternak S, et al. The B73 maize genome: Complexity, diversity, and dynamics. Science (80- ). 2009;326:1112–5. 10.1126/science.1178534 19965430

[81] Danecek P, Auton A, Abecasis G, Albers CA, Banks E, DePristo MA, et al. The variant call format and VCFtools. Bioinformatics. 2011;27:2156–8. 21653522 10.1093/bioinformatics/btr330 PMC3137218

[82] Barrett JC, Fry B, Maller J, Daly MJ. Haploview: Analysis and visualization of LD and haplotype maps. Bioinformatics. 2005;21:263–5. 15297300 10.1093/bioinformatics/bth457

[83] Zhang R, Jia G, Diao X. geneHapR: an R package for gene haplotypic statistics and visualization. BMC Bioinform. 2023;24:199. 10.1186/s12859-023-05318-9 PMC10186671 37189023

[84] Gabriel SB, Schaffner SF, Nguyen H, Moore JM, Roy J, Blumenstiel B, et al. The structure of haplotype blocks in the human genome. Science (80- ). 2002;296:2225–9. 10.1126/science.1069424 12029063

[85] Endelman JB. Ridge Regression and Other Kernels for Genomic Selection with R Package rrBLUP. Plant Genome. 2011;4:250–5.

[86] Wickham H. ggplot2. Springer-V. New York, NY: Springer New York; 2009.

[87] Cairns JE, Prasanna BM. Developing and deploying climate-resilient maize varieties in the developing world. Curr Opin Plant Biol. 2018;45:226–30. 29779966 10.1016/j.pbi.2018.05.004 PMC6250980

[88] Wang Y, Guo H, Wu X, Wang J, Li H, Zhang R. Transcriptomic and physiological responses of contrasting maize genotypes to drought stress. Front Plant Sci. 2022;13:928897. 35991451 10.3389/fpls.2022.928897 PMC9381927

[89] Blancon J, Buet C, Dubreuil P, Tixier MH, Baret F, Praud S. Maize green leaf area index dynamics: genetic basis of a new secondary trait for grain yield in optimal and drought conditions. Theor Appl Genet. 2024;137:68. 38441678 10.1007/s00122-024-04572-6 PMC10914915

[90] Cairns JE, Crossa J, Zaidi PH, Grudloyma P, Sanchez C, Luis Araus J, et al. Identification of drought, heat, and combined drought and heat tolerant donors in maize. Crop Sci. 2013;53:1335–46.

[91] Trachsel S, Leyva M, Lopez M, Suarez EA, Mendoza A, Montiel NG, et al. Identification of tropical maize germplasm with tolerance to drought, nitrogen deficiency, and combined heat and drought stresses. Crop Sci. 2016;56:3031–45.

[92] Rezende WS, Beyene Y, Mugo S, Ndou E, Gowda M, Sserumaga JP, et al. Performance and yield stability of maize hybrids in stress-prone environments in eastern Africa. Crop J. 2020;8:107–18.

[93] Beyene Y, Mugo S, Semagn K, Asea G, Trevisan W, Tarekegne A, et al. Genetic distance among doubled haploid maize lines and their testcross performance under drought stress and non-stress conditions. Euphytica. 2013;192:379–92.

[94] Chapman SC, Edmeades GO. Selection improves drought tolerance in tropical maize populations: II Direct and correlated responses among secondary traits. Crop Sci. 1999;39:1315–24.

[95] Ndlovu N, Gowda M, Beyene Y, Chaikam V, Nzuve FM, Makumbi D, et al. Genomic loci associated with grain yield under well-watered and water-stressed conditions in multiple bi-parental maize populations. Front Sustain Food Syst. 2024;8:1391989.

[96] Bolaños J, Edmeades GO. The importance of the anthesis-silking interval in breeding for drought tolerance in tropical maize. F Crop Res. 1996;48:65–80.

[97] Badu-Apraku B, Akinwale RO, Franco J, Oyekunle M. Assessment of reliability of secondary traits in selecting for improved grain yield in drought and low-nitrogen environments. Crop Sci. 2012;52:2050–62.

[98] Zhang X, Ren Z, Luo B, Zhong H, Ma P, Zhang H, et al. Genetic architecture of maize yield traits dissected by QTL mapping and GWAS in maize. Crop J. 2022;10:436–46.

[99] Clark RM, Linton E, Messing J, Doebley JF. Pattern of diversity in the genomic region near the maize domestication gene tb1. Proc Natl Acad Sci U S A. 2004;101:700–7. 14701910 10.1073/pnas.2237049100 PMC321743

[100] Rashid Z, Sofi M, Harlapur SI, Kachapur RM, Dar ZA, Singh PK, et al. Genome-wide association studies in tropical maize germplasm reveal novel and known genomic regions for resistance to Northern corn leaf blight. Sci Rep. 2020;10:1–16. 33319847 10.1038/s41598-020-78928-5 PMC7738672

[101] Lu Y, Shah T, Hao Z, Taba S, Zhang S, Gao S, et al. Comparative SNP and haplotype analysis reveals a higher genetic diversity and rapider LD decay in tropical than temperate germplasm in maize. PLoS One. 2011;6:e24861. 21949770 10.1371/journal.pone.0024861 PMC3174237

[102] Xiong X, Li J, Su P, Duan H, Sun L, Xu S, et al. Genetic dissection of maize (Zea mays L.) chlorophyll content using multi-locus genome-wide association studies. BMC Genomics. 2023;24:384. 37430212 10.1186/s12864-023-09504-0 PMC10332058

[103] Zhou G, Zhu Q, Mao Y, Chen G, Xue L, Lu H, et al. Multi-Locus Genome-Wide Association Study and Genomic Selection of Kernel Moisture Content at the Harvest Stage in Maize. Front Plant Sci. 2021;12:1–13. 10.3389/fpls.2021.697688 PMC8299107 34305987

[104] Wen YJ, Wu X, Wang S, Han L, Shen B, Wang Y, et al. Identification of QTN-by-environment interactions for yield related traits in maize under multiple abiotic stresses. Front Plant Sci. 2023;14:1050313. 36875585 10.3389/fpls.2023.1050313 PMC9975332

[105] Grotewold E, Gray J. Maize transcription factors. Handb Maize Genet Genomics. 2009;II:693–713.

[106] Ningning Z, Binbin L, Fan Y, Jianzhong C, Yuqian Z, Yejian W, et al. Molecular mechanisms of drought resistance using genome-wide association mapping in maize (Zea mays L.). BMC Plant Biol. 2023;23:1–17. 37803273 10.1186/s12870-023-04489-0 PMC10557160

[107] Zhu Y, Liu Y, Zhou K, Tian C, Aslam M, Zhang B, et al. Overexpression of ZmEREBP60 enhances drought tolerance in maize. J Plant Physiol. 2022;275:153763. 35839657 10.1016/j.jplph.2022.153763

[108] Qi H, Liang K, Ke Y, Wang J, Yang P, Yu F, et al. Advances of Apetala2/Ethylene Response Factors in Regulating Development and Stress Response in Maize. Int J Mol Sci. 2023;24:5416. 36982510 10.3390/ijms24065416 PMC10049130

[109] Zhang J, Liao J, Ling Q, Xi Y, Qian Y. Genome-wide identification and expression profiling analysis of maize AP2/ERF superfamily genes reveal essential roles in abiotic stress tolerance. BMC Genomics. 2022;23:125. 35151253 10.1186/s12864-022-08345-7 PMC8841118

[110] Farmer LM, Book AJ, Lee KH, Lin YL, Fu H, Vierstraa RD. The RAD23 family provides an essential connection between the 26S proteasome and ubiquitylated proteins in Arabidopsis. Plant Cell. 2010;22:124–42. 20086187 10.1105/tpc.109.072660 PMC2828702

[111] Doroodian P, Hua Z. The ubiquitin switch in plant stress response. Plants. 2021;10:1–21. 10.3390/plants10020246 PMC7911189 33514032

[112] Wang X, An Y, Xu P, Xiao J. Functioning of PPR Proteins in Organelle RNA Metabolism and Chloroplast Biogenesis. Front Plant Sci. 2021;12:1–8. 10.3389/fpls.2021.627501 PMC7900629 33633768

[113] Xing H, Fu X, Yang C, Tang X, Guo L, Li C, et al. Genome-wide investigation of pentatricopeptide repeat gene family in poplar and their expression analysis in response to biotic and abiotic stresses. Sci Rep. 2018;8:1–9. 29434322 10.1038/s41598-018-21269-1 PMC5809412

[114] Zhu X, Ze M, Yin J, Chern M, Wang M, Zhang X, et al. A phosphofructokinase B-type carbohydrate kinase family protein, PFKB1, is essential for chloroplast development at early seedling stage in rice. Plant Sci. 2020;290:110295. 31779907 10.1016/j.plantsci.2019.110295

[115] Peng Li , Zhaoxia Li GX and JZ*. Trihelix Transcription Factor ZmThx20 Is Required for Kernel Development in Maize. Int J Mol Sci. 2021;22:12137. 10.3390/ijms222212137 PMC8624104 34830019

[116] Zhao D, Gao F, Guan P, Gao J, Guo Z, Guo J, et al. Identification and analysis of differentially expressed trihelix genes in maize (Zea mays) under abiotic stresses. PeerJ. 2023;11:e15312. 37151290 10.7717/peerj.15312 PMC10158769

[117] Riggs JW, Rockwell NC, Cavales PC, Callis J. Identification of the plant ribokinase and discovery of a role for Arabidopsis Ribokinase in nucleoside metabolism. J Biol Chem. 2016;291:22572–82. 27601466 10.1074/jbc.M116.754689 PMC5077194

[118] Yang J, Tang Z, Yang W, Huang Q, Wang Y, Huang M, et al. Genome-wide characterization and identification of Trihelix transcription factors and expression profiling in response to abiotic stresses in Chinese Willow (Salix matsudana Koidz). Front Plant Sci. 2023;14:1125519. 36938039 10.3389/fpls.2023.1125519 PMC10020544

[119] Ran F, Wang Y, Jiang F, Yin X, Bi Y, Shaw RK, et al. Studies on Candidate Genes Related to Flowering Time in a Multiparent Population of Maize Derived from Tropical and Temperate Germplasm. Plants. 2024;13:1032. 38611561 10.3390/plants13071032 PMC11013272

[120] Khan SU, Zheng Y, Chachar Z, Zhang X, Zhou G, Zong N, et al. Dissection of Maize Drought Tolerance at the Flowering Stage Using Genome-Wide Association Studies. Genes (Basel). 2022;13:564. 35456369 10.3390/genes13040564 PMC9031386

[121] Lu Y, Zhang S, Shah T, Xie C, Hao Z, Li X, et al. Joint linkage-linkage disequilibrium mapping is a powerful approach to detecting quantitative trait loci underlying drought tolerance in maize. Proc Natl Acad Sci U S A. 2010;107:19585–90. 20974948 10.1073/pnas.1006105107 PMC2984198

[122] Raunser S, Magnani R, Huang Z, Houtz RL, Trievel RC, Penczek PA, et al. Rubisco in complex with Rubisco large subunit methyltransferase. Proc Natl Acad Sci U S A. 2009;106:3160–5. 19208805 10.1073/pnas.0810563106 PMC2638739

[123] Mininno M, Brugière S, Pautre V, Gilgen A, Ma S, Ferro M, et al. Characterization of chloroplastic fructose 1,6-bisphosphate aldolases as lysine-methylated proteins in plants. J Biol Chem. 2012;287:21034–44. 22547063 10.1074/jbc.M112.359976 PMC3375527

[124] Li J, Zhang Y, Gao Z, Xu X, Wang Y, Lin Y, et al. Plant U-box E3 ligases PUB25 and PUB26 control organ growth in Arabidopsis. New Phytol. 2021;229:403–13. 32810874 10.1111/nph.16885

[125] Trujillo M. News from the PUB: Plant U-box type E3 ubiquitin ligases. J Exp Bot. 2018;69:371–84. 29237060 10.1093/jxb/erx411

[126] Liu Y, Li C, Qin A, Deng W, Chen R, Yu H, et al. Genome-wide identification and transcriptome profiling expression analysis of the U-box E3 ubiquitin ligase gene family related to abiotic stress in maize (Zea mays L). BMC Genomics. 2024;25:132. 38302871 10.1186/s12864-024-10040-8 PMC10832145

[127] Jia T, Ge Q, Zhang S, Zhang Z, Liu A, Fan S, et al. UDP-Glucose Dehydrogenases: Identification, Expression, and Function Analyses in Upland Cotton (Gossypium hirsutum). Front Genet. 2021;11:597890. 33505427 10.3389/fgene.2020.597890 PMC7831515

[128] Kärkönen A, Murigneux A, Martinant JP, Pepey E, Tatout C, Dudley BJ, et al. UDP-glucose dehydrogenases of maize: A role in cell wall pentose biosynthesis. Biochem J. 2005;391:409–15. 15969652 10.1042/BJ20050800 PMC1276940

[129] Wu H, Bai B, Lu X, Li H. A gibberellin-deficient maize mutant exhibits altered plant height, stem strength and drought tolerance. Plant Cell Rep. 2023;42:1687–99. 37479884 10.1007/s00299-023-03054-1

[130] Martín-Trillo M, Cubas P. TCP genes: a family snapshot ten years later. Trends Plant Sci. 2010;15:31–9. 19963426 10.1016/j.tplants.2009.11.003

[131] Lan T, Xiong W, Chen X, Mo B, Tang G. Plant cytoplasmic ribosomal proteins: an update on classification, nomenclature, evolution and resources. Plant J. 2022;110:292–318. 35000252 10.1111/tpj.15667

[132] Li Y, Liu F, Li P, Wang T, Zheng C, Hou B. An Arabidopsis Cytokinin-Modifying Glycosyltransferase UGT76C2 Improves Drought and Salt Tolerance in Rice. Front Plant Sci. 2020;11:1–15. 33224159 10.3389/fpls.2020.560696 PMC7674613

[133] Jia H, Li M, Li W, Liu L, Jian Y, Yang Z, et al. A serine/threonine protein kinase encoding gene KERNEL NUMBER PER ROW6 regulates maize grain yield. Nat Commun. 2020;11:988. 32080171 10.1038/s41467-020-14746-7 PMC7033126

[134] Shohat H, Eliaz NI, Weiss D. Gibberellin in tomato: metabolism, signaling and role in drought responses. Mol Hortic. 2021;1:15. 37789477 10.1186/s43897-021-00019-4 PMC10515025

[135] Li S. The Arabidopsis thaliana TCP transcription factors: A broadening horizon beyond development. Plant Signaling & Behavior. 2015;10:e1044192. 10.1080/15592324.2015.1044192 PMC4622585 26039357

[136] Liu Y, Chen Z, Zhang C, Guo J, Liu Q, Yin Y, et al. Gene editing of ZmGA20ox3 improves plant architecture and drought tolerance in maize. Plant Cell Rep. 2024;43:18. 10.1007/s00299-023-03090-x 38148416

[137] Jiao P, Liu T, Zhao C, Fei J, Guan S, Ma Y. ZmTCP14, a TCP transcription factor, modulates drought stress response in Zea mays L. Environ Exp Bot. 2023;208:105232.

[138] Ding S, Cai Z, Du H, Wang H. Genome-wide analysis of TCP family genes in Zea mays l Identified a role for ZmTCP42 in drought tolerance. Int J Mol Sci. 2019;20:2762. 31195663 10.3390/ijms20112762 PMC6600213

[139] Takemiya A, Sugiyama N, Fujimoto H, Tsutsumi T, Yamauchi S, Hiyama A, et al. Phosphorylation of BLUS1 kinase by phototropins is a primary step in stomatal opening. Nat Commun. 2013;4:2094. 23811955 10.1038/ncomms3094

[140] Li C, Chen G, Mishina K, Yamaji N, Ma JF, Yukuhiro F, et al. A GDSL-motif esterase/acyltransferase/lipase is responsible for leaf water retention in barley. Plant Direct. 2017;1:e00025. 31245672 10.1002/pld3.25 PMC6508521

[141] Shen G, Sun W, Chen Z, Shi L, Hong J, Shi J. Plant GDSL Esterases/Lipases: Evolutionary, Physiological and Molecular Functions in Plant Development. Plants. 2022;11:468. 35214802 10.3390/plants11040468 PMC8880598

[142] Su HG, Zhang XH, Wang TT, Wei WL, Wang YX, Chen J, et al. Genome-Wide Identification, Evolution, and Expression of GDSL-Type Esterase/Lipase Gene Family in Soybean. Front Plant Sci. 2020;11:726. 32670311 10.3389/fpls.2020.00726 PMC7332888

[143] Li Z, Ahammed GJ. Plant stress response and adaptation via anthocyanins: A review. Plant Stress. 2023;10:100230.

[144] Lu N, Jun JH, Li Y, Dixon RA. An unconventional proanthocyanidin pathway in maize. Nat Commun. 2023;14:4349. 37468488 10.1038/s41467-023-40014-5 PMC10356931

[145] Zhang Q, Liu H, Wu X, Wang W. Identification of drought tolerant mechanisms in a drought-tolerant maize mutant based on physiological, biochemical and transcriptomic analyses. BMC Plant Biol. 2020;20:1–14. 32620139 10.1186/s12870-020-02526-w PMC7350183

[146] Kushwaha HR, Joshi R, Pareek A, Singla-Pareek SL. MATH-domain family shows response toward abiotic stress in arabidopsis and rice. Front Plant Sci. 2016;7:923. 27446153 10.3389/fpls.2016.00923 PMC4923191

[147] Cai G, Zang Y, Wang Z, Liu S, Wang G. Arabidopsis BTB-A2s Play a Key Role in Drought Stress. Biology (Basel). 2024;13:561. 39194499 10.3390/biology13080561 PMC11351226

[148] Zeng T, Meng Z, Yue R, Lu S, Li W, Li W, et al. Genome wide association analysis for yield related traits in maize. BMC Plant Biol. 2022;22:1–11. 36127632 10.1186/s12870-022-03812-5 PMC9490995

[149] Guo J, Sun B, He H, Zhang Y, Tian H, Wang B. Current understanding of bhlh transcription factors in plant abiotic stress tolerance. International J Mol Sci. 2021;22:4921. 10.3390/ijms22094921 PMC8125693 34066424

[150] Zhang T, Lv W, Zhang H, Ma L, Li P, Ge L, et al. Genome-wide analysis of the basic Helix-Loop-Helix (bHLH) transcription factor family in maize. BMC Plant Biol. 2018;18:1–4. 30326829 10.1186/s12870-018-1441-z PMC6192367

[151] Nan GL, Teng C, Fernandes J, O’Connor L, Meyers BC, Walbot V. A cascade of bHLH-regulated pathways programs maize anther development. Plant Cell. 2022;34:1207–25. 35018475 10.1093/plcell/koac007 PMC8972316

[152] He Z, Zhong J, Sun X, Wang B, Terzaghi W, Dai M. The maize ABA receptors ZmPYl8, 9, and 12 facilitate plant drought resistance. Front Plant Sci. 2018;9:422. 29670640 10.3389/fpls.2018.00422 PMC5893742

[153] Niu L, Liu L, Wang W. Digging for Stress-Responsive Cell Wall Proteins for Developing Stress-Resistant Maize. Front Plant Sci. 2020;11:576385. 33101346 10.3389/fpls.2020.576385 PMC7546335

[154] Song N, Xu Z, Wang J, Qin Q, Jiang H, Si W, et al. Genome-wide analysis of maize CONSTANS-LIKE gene family and expression profiling under light/dark and abscisic acid treatment. Gene. 2018;673:1–11. 29908279 10.1016/j.gene.2018.06.032

[155] Wang F, Han T, Jeffrey CZ. Circadian and photoperiodic regulation of the vegetative to reproductive transition in plants. Commun Biol. 2024;7:1–11. 38755402 10.1038/s42003-024-06275-6 PMC11098820

[156] Bhat JA, Yu D, Bohra A, Ganie SA, Varshney RK. Features and applications of haplotypes in crop breeding. Commun Biol. 2021;4:1–12. 34737387 10.1038/s42003-021-02782-y PMC8568931

[157] Beyene Y, Gowda M, Olsen M, Robbins KR, Pérez-Rodríguez P, Alvarado G, et al. Empirical Comparison of Tropical Maize Hybrids Selected Through Genomic and Phenotypic Selections. Front Plant Sci. 2019;10:1502. 31824533 10.3389/fpls.2019.01502 PMC6883373

[158] Ertiro BT, Labuschagne M, Olsen M, Das B, Prasanna BM, Gowda M. Genetic Dissection of Nitrogen Use Efficiency in Tropical Maize Through Genome-Wide Association and Genomic Prediction. Front Plant Sci. 2020;11:1–16. 32411159 10.3389/fpls.2020.00474 PMC7198882

