# Supplementary figures and images for: Genome-wide association mapping and genomic prediction analyses reveal the genetic architecture of grain yield and agronomic traits under drought and optimum conditions in maize
Source: BMC Plant Biol. 2025 Feb 1;25:135. doi: 10.1186/s12870-025-06135-3 (PMC11786572; doi:10.1186/s12870-025-06135-3)

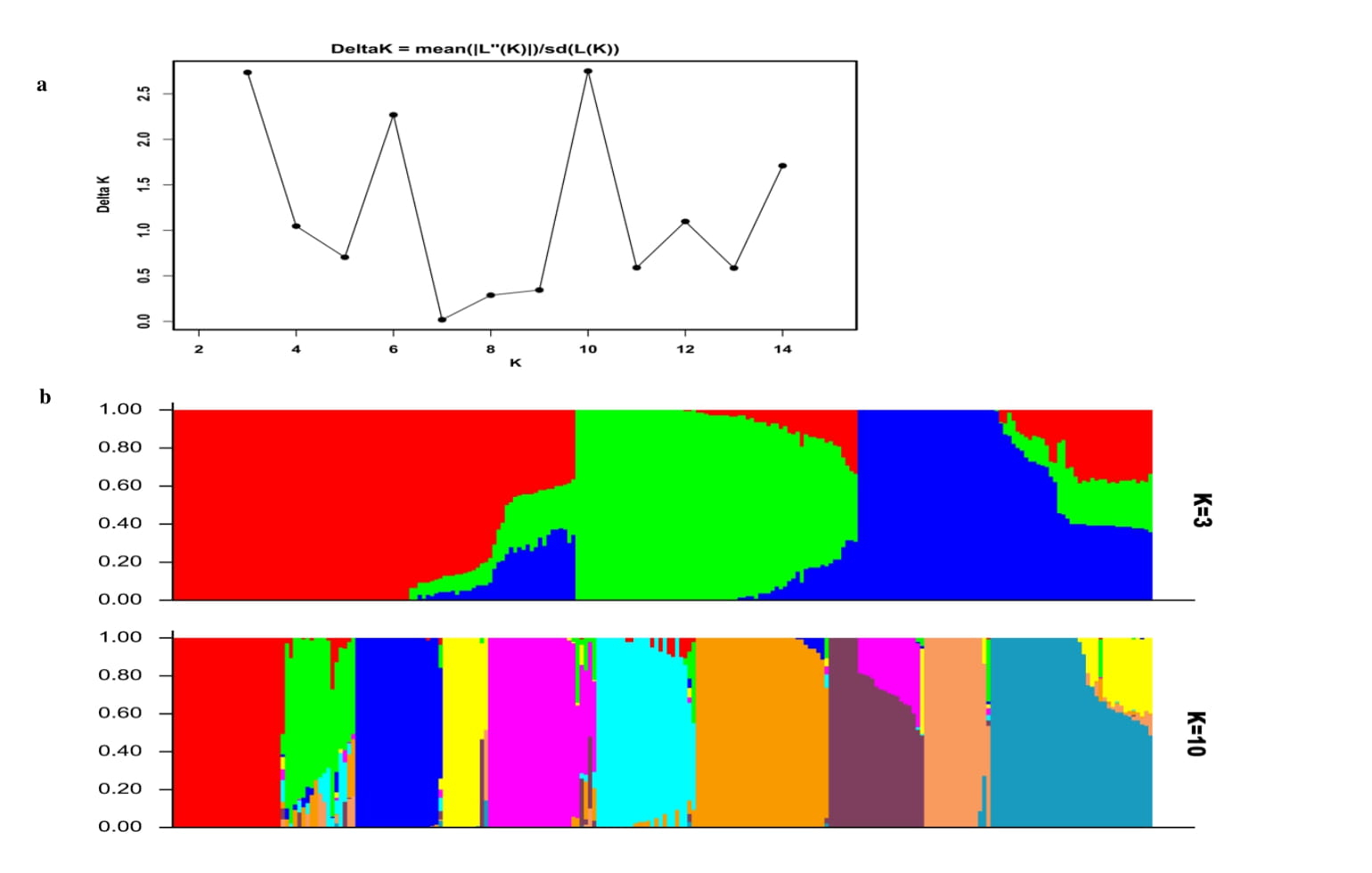

Supplement: Supplementary file 1 — Supplementary Material 1: Supplementary Figure S1. Population structure analyses of 236 maize inbred lines based on 215,542 SNPs : (a) Evanno plot of the number of clusters (K) against delta K to determine the optimum number of K; (b) graphical representation of the 236 lines at K = 3 to K = 10. Each individual is shown as a vertical line divided into K colored segments, with segment lengths indicating the estimated probability of membership to each cluster. [file 12870_2025_6135_MOESM1_ESM.jpg]

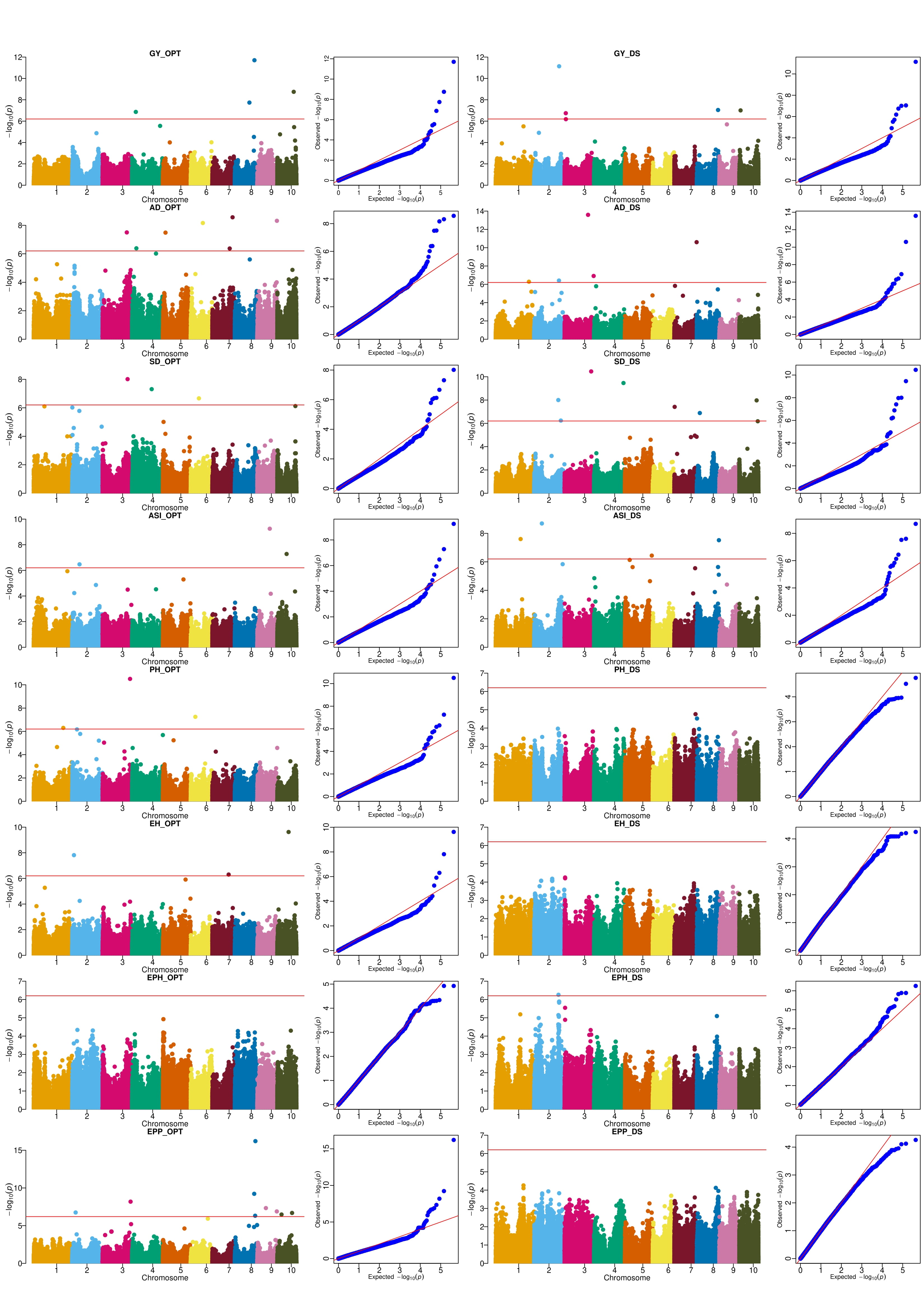

Supplement: Supplementary file 2 — Supplementary Material 2: Supplementary Figure S2. Farm CPU-based Manhattan and Q-Q plots of genome-wide association study (GWAS) on eight traits evaluated under optimum (_OPT) and drought (_DS) environmental conditions. The − log10(p) values on the Y-axis in the Manhattan plot represent grain yield (GY), Days to 50 % anthesis, (AD), Days to 50 % silking (SD), Anthesis-Silking Interval (ASI), Plant height (PH), Ear height (EH), Ear position (EPO) and Ear per plant (EPP) plotted against chromosome position on X-axis. The red and blue solid horizontal lines in the Manhattan plots represent the genome-wide (− log10 (p) =6.2). The quantile—quantile plots represent observed against the expected −log10 (p). [file 12870_2025_6135_MOESM2_ESM.jpg]

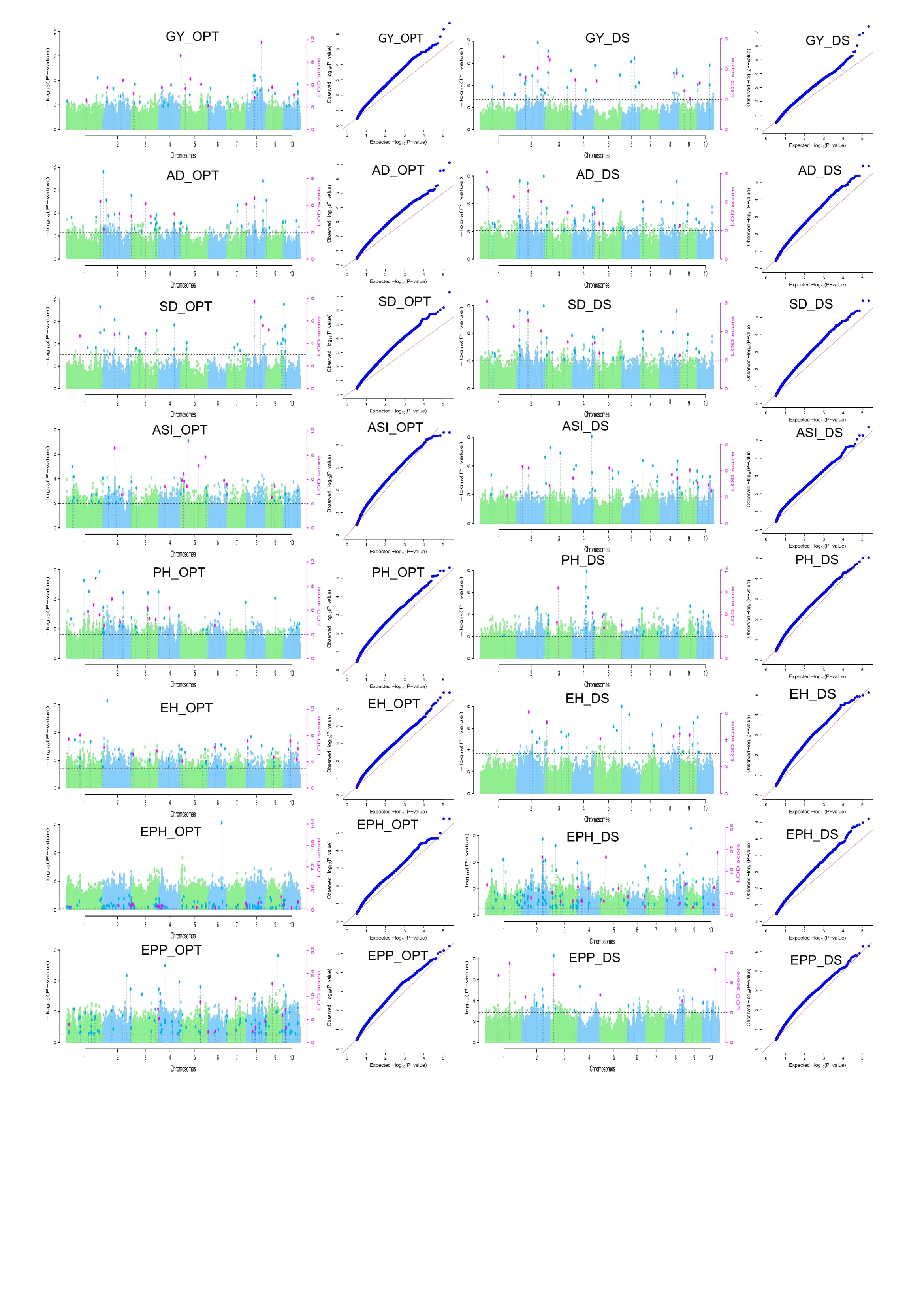

Supplement: Supplementary file 3 — Supplementary Material 3: Supplementary Figure S3. Manhattan and Q-Q plots of genome-wide association study (GWAS) on eight traits evaluated under optimum (OPT) and drought (_DS) environmental conditions for six GWAS models. The pink dots above the threshold indicates significant QTNs identified by more than one ML-GWAS models, while green and blue dots above the threshold represent significant QTNs identified by a single ML-GWAS model . The black horizontal dashed line indicates the genome-wide significance threshold, corresponding to a −log10 (p) value of a LOD score ≥ 3.0 for ML-GWAS models. [file 12870_2025_6135_MOESM3_ESM.jpg]
